# Simple, Reliable Protocol for High-Yield Solubilization of Seedless Amyloid-β Monomer

**DOI:** 10.1021/acschemneuro.2c00411

**Published:** 2022-12-13

**Authors:** Alexander I. P. Taylor, Peter J. Davis, Liam D. Aubrey, Joshua B. R. White, Zoe N. Parton, Rosemary A. Staniforth

**Affiliations:** School of Biosciences, University of Sheffield, SheffieldS10 2TN, United Kingdom

**Keywords:** Alzheimer’s disease, amyloidogenesis, protein preparation, flow fractionation, multiangle light scattering, peptides

## Abstract

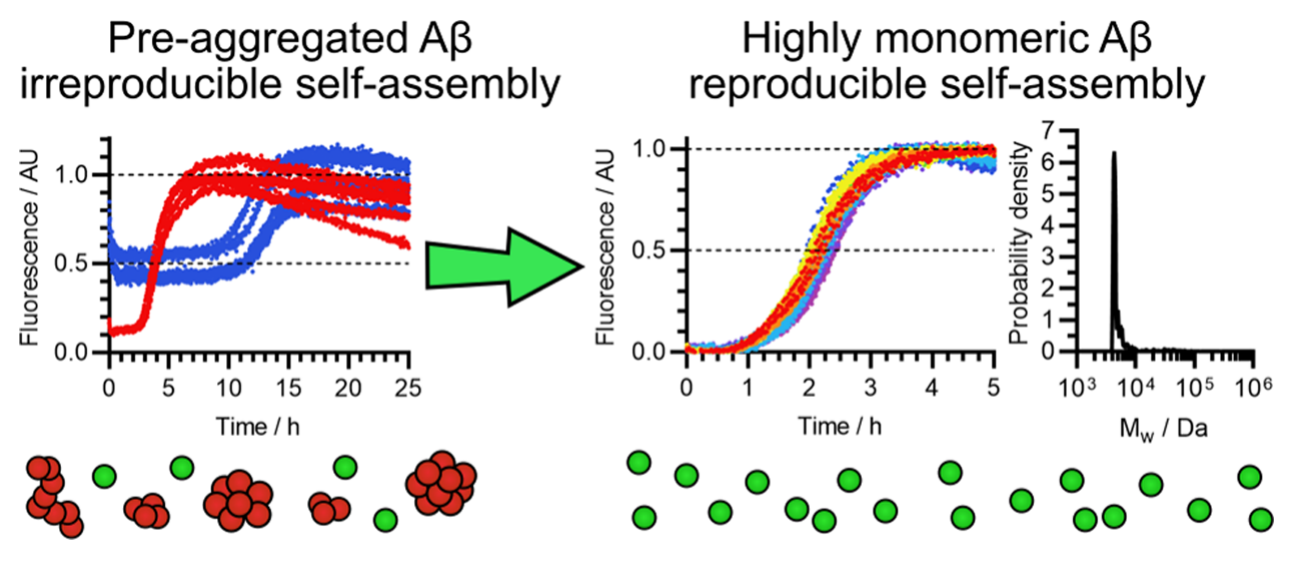

Self-assembly of the amyloid-β (Aβ) peptide to form toxic oligomers and fibrils is a key causal event in the onset of Alzheimer’s disease, and Aβ is the focus of intense research in neuroscience, biophysics, and structural biology aimed at therapeutic development. Due to its rapid self-assembly and extreme sensitivity to aggregation conditions, preparation of seedless, reproducible Aβ solutions is highly challenging, and there are serious ongoing issues with consistency in the literature. In this paper, we use a liquid-phase separation technique, asymmetric flow field-flow fractionation with multiangle light scattering (AF4-MALS), to develop and validate a simple, effective, economical method for re-solubilization and quality control of purified, lyophilized Aβ samples. Our findings were obtained with recombinant peptide but are physicochemical in nature and thus highly relevant to synthetic peptide. We show that much of the variability in the literature stems from the inability of overly mild solvent treatments to produce consistently monomeric preparations and is rectified by a protocol involving high-pH (>12) dissolution, sonication, and rapid freezing to prevent modification. Aβ treated in this manner is chemically stable, can be stored over long timescales at −80 °C, and exhibits remarkably consistent self-assembly behavior when returned to near-neutral pH. These preparations are highly monomeric, seedless, and do not require additional rounds of size exclusion, eliminating the need for this costly procedure and increasing the flexibility of use. We propose that our improved protocol is the simplest, fastest, and most effective way to solubilize Aβ from diverse sources for sensitive self-assembly and toxicity assays.

## 1 Introduction

Amyloid-β (Aβ) is a disordered peptide that plays a key role in the onset of Alzheimer’s disease (AD).^1−6^*In vivo*, Aβ self-assembles to form amyloid fibrils, ribbon-like fibers of aggregated protein that have a characteristic “cross-β” structure, and are the primary constituents of the senile plaques found in AD brains.^1−3^ In addition, Aβ forms a diverse range of smaller, transient, and typically less structurally ordered assemblies, usually termed oligomers or protofibrils, which serve as intermediates or off-pathway byproducts of amyloid formation.^7−17^ Many Aβ oligomers are highly neurotoxic, causing disruption or damage to lipid membranes^18−25^ and the activation of signaling proteins such as glycogen synthase kinase-3β (GSK-3β)^10^ and *N*-methyl-d-aspartate receptor (NMDAR).^26^ In turn, this leads to neuronal calcium dyshomeostasis,^20,27^ impaired long-term potentiation,^9,28,29^ mitochondrial dysfunction,^30,31^ and phosphorylation and altered activity of the Tau protein,^32−34^ another key causative agent in AD pathophysiology.^35,36^ In addition, mature amyloid fibrils catalyze the formation of toxic pre-fibrillar oligomers by a process termed secondary nucleation, creating a positive feedback loop between oligomer and fibril formation that further exacerbates aggregation and toxicity.^15,16,25,37^ Thus, Aβ has an important role in mediating the onset and progression of AD and is a major target for therapeutic development.

The development of agents that inhibit Aβ aggregation and neurotoxicity requires a high standard of experimental reproducibility and control. Therefore, it is essential that Aβ preparation protocols yield well-defined, highly monomeric solutions with minimal seeding or chemical modification. However, despite more than 50 years of intense experimental research, there is still no consensus on how to achieve this, and, consequently, the reproducibility of the literature remains poor. For example, different groups using apparently similar conditions have reported fibrillization half-times spanning several orders of magnitude,^38−41^ and many have also reported considerable variation between repeat experiments.^40,42,43^ Rate differences directly reflect the presence of uncharacterized contaminants and chemical modifications, and call into question the validity of many results.

The re-solubilization of lyophilized Aβ samples is a major source of avoidable, mechanistically impactful variation. Both synthetic and recombinant preparations are typically lyophilized for storage, and re-solubilized immediately before use. Protocols for re-solubilizing Aβ are diverse, making use of a broad range of solvents such as hexafluoroisopropanol (HFIP),^7,44−52^ NaOH,^41,46−50,53,54^ and NH_3_OH,^7,52,55−57^ with different concentrations and dissolution times. In addition, they vary widely in the inclusion of additional steps such as sonication, filtration, or further rounds of size exclusion chromatography (SEC).^7,41,45,47,54,55,58−68^ However, the extreme sensitivity of Aβ to its self-assembly conditions means that solubilization-dependent factors such as chemical modification, seeding, off-pathway aggregation, and the presence of residual co-solutes can all strongly affect the self-assembly process. Thus, the diversity of re-solubilization procedures is a likely contributor to the poor reproducibility of the experimental literature.

In this paper, we use a novel fractionation method, asymmetric flow field-flow fractionation (AF4),^69^ to develop and validate a standardized protocol for re-solubilization of Aβ samples. While previous studies have recommended dilute base treatments for re-solubilization (e.g., 1–10 mM NaOH),^41,46,48,53^ we show that the resulting sample pH (typically <11, following partial neutralization) is insufficient to fully monomerize the peptide or prevent seeding or off-pathway aggregation. Instead, we advocate the use of more concentrated base (50 mM NaOH, resulting in pH ≈ 12.5), coupled with sonication and storage at −80 °C, to fully solubilize the peptide and ensure long-term stability. This approach produces highly monomeric samples that are free from fibril seeds, off-pathway aggregates, or detectable chemical modifications such as truncation or deamidation, and allows recovery of close to 100% of the peptide as monomer or rapidly equilibrating concentration-induced small oligomers. Peptide prepared in this way is stable for experimentally convenient timescales at room temperature, and a period of months or possibly years at −80 °C. Controlled self-assembly can easily be initiated by dilution into a pre-adjusted aggregation buffer of the user’s choice, and kinetic assays conducted under standard conditions reveal concentration-dependent, sigmoidal, classically unseeded kinetics. Moreover, while many groups use additional purification steps such as SEC to remove pre-formed aggregates,^7,55,62,63,65−67^ we show that these steps are only necessary for impure, aggregated, or improperly re-solubilized peptide, and do not affect the composition or kinetics of purified samples that have been treated with 50 mM NaOH. Therefore, these additional steps, which result in considerable expenditure of peptide and severely restrict the timescale and Aβ concentration of downstream experiments, can be relegated to control experiments so long as our recommended re-solubilization procedure is followed. Based on these findings, we recommend a standardized procedure for re-solubilization and quality control of purified, lyophilized Aβ from in-house recombinant, commercial recombinant, and synthetic sources. We expect our improved protocol to substantially improve solubilization yield, experimental consistency, and flexibility for the vast majority of users.

## 2 Results and Discussion

### 2.1 Fractionation Method for Efficient Quality Control

To develop an improved solubilization protocol, we required a sensitive analytical technique capable of detecting even small quantities of fibril seeds and resolving samples with a broad range of particle sizes, including monomers, oligomers, amyloid fibrils, and amorphous aggregates. While SEC and gel electrophoresis are commonly used to validate preparations, they do not allow separation of amyloid fibrils or other large aggregates, which are too large to pass through the gel or column matrix. As a result, the presence of these species tends to be obscured. In addition, SEC suffers from limited scope for optimization, gel electrophoresis requires additional cross-linking to stabilize small oligomers, and both techniques expose a large surface area, which has the potential to interact with Aβ, altering the self-assembly process. Alternatively, solution-state NMR is sometimes used for validation, but NMR is also unable to detect large aggregates, and struggles to resolve monomers from oligomers in mixed samples, particularly when the latter is at low abundance.

**Figure 1:**
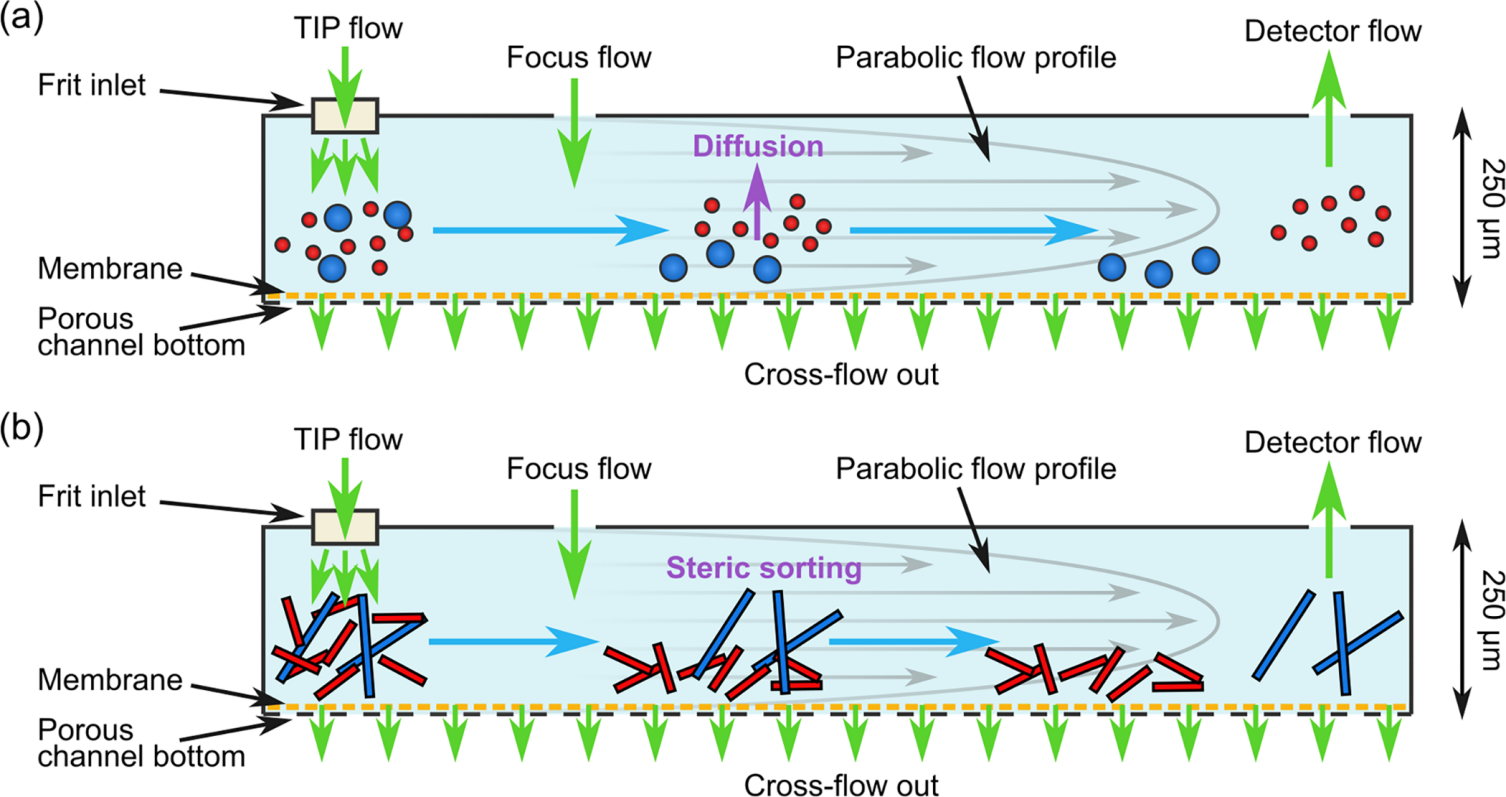
Schematic of sample separation by AF4 with a frit inlet (FI) channel. (a) Operation in normal mode. (b) Operation in steric mode. In both schematics, green arrows indicate flows in and out of the channel, blue arrows indicate the general direction of flow within the channel, and gray arrows represent the parabolic flow profile, with flow faster toward the center of the channel and slower toward the edges.

Asymmetric flow field-flow fractionation (AF4) is a powerful analytical technique capable of separating particles with a broad range of sizes, from 1 nm to 50 μm.^69,70^ As depicted in Figure 1, AF4 circumvents the above issues using a system of orthogonal flows to sort particles by hydrodynamic radius, *R*_h_, and a laminar flow gradient to translate this into different elution times. As a result, separation is achieved in the liquid phase and is highly optimizable. Samples are then detected and characterized by multiangle light scattering (MALS) and UV absorbance spectrometry. In the normal mode of AF4, shown in Figure 1a, the sample flows from one end of a channel to the other, and a cross-flow orthogonal to the direction of elution pulls the sample toward a semipermeable membrane at the channel base. Particles with a smaller *R*_h_ diffuse more effectively against this cross-flow, and occupy a higher, more central position in the channel; particles with a larger *R*_h_ diffuse less effectively against this cross-flow, and accumulate closer to the membrane. A parabolic flow profile means that the elution buffer flows faster closer to the center of the channel so that the smaller particles elute earlier and the larger particles elute later.^69,70^ Alternatively, in the steric mode of elution, shown in Figure 1b, the accumulation of particularly large particles (≳500 nm) close to the membrane is limited by steric occlusion, resulting in a reversal of elution order.^70,71^ In practice, due to the broad size range of our samples, we observe a mixed elution mode so that large fibril-sized aggregates elute first, followed by monomer-sized aggregates, oligomers, and then aggregates with a size equivalent to small amyloid fibrils. AF4 lacks the invasive solid phase of SEC, is more finely tunable due to the capacity to optimize flow profiles, and allows recovery of large particles that would be lost in a column matrix. By coupling this technique with sensitive UV absorbance and multiangle light scattering (MALS) detection, both small and large aggregates can be resolved even at low concentrations.

### 2.2 Widely Used Aβ Solubilization Protocols Result in Unreliable Self-Assembly Kinetics Due to Incomplete Monomerization

Aβ samples that are stored in a lyophilized form are often re-solubilized by treatment with a dilute base such as 1–10 mM NaOH or 0.02–1% NH_4_OH.^41,46,48,53,56,57^ These treatments would be expected to give a pH of 10.6–12.0, in the absence of any partial neutralization of the solvent. However, fluorescence-based assays have indicated that Aβ(1-42) still undergoes limited aggregation at pH 11.0, on an approximate timescale of several hundred seconds, and the threshold for complete elimination of self-assembly appears to be somewhere in the pH 11.0–12.0 range.^72^ In addition, many studies do not report checking the final pH of Aβ samples following re-solubilization, raising the question of whether partial neutralization would result in a lower pH than expected, exacerbating the issue. As discussed in Section 2.1, prevailing methods that are often used to validate Aβ preparations struggle to detect contaminating aggregates that are large, or present at low abundance. Therefore, we hypothesized that incomplete monomerization and/or partial pre-aggregation might be a common feature of Aβ samples re-solubilized with a dilute base, and this could be responsible for a significant portion of the inconsistency in the literature.

To test our hypothesis, we investigated the effectiveness of re-solubilizing 1 mg/mL (222 μM) Aβ(1-42) in 10 mM NaOH. This would be expected to give a final pH of 12.0, excluding any neutralization of the NaOH solvent, and thus reflects the upper end of the aforementioned pH range. Two protocols were used: the first involved monomerization by sonication for 30 min in HFIP, followed by aliquoting, re-lyophilization, and dissolution in 10 mM NaOH at the point of use; and the second involved sonication for 30 min in 10 mM NaOH, followed by aliquoting, freezing, and thawing at the point of use. While the former was based on the protocol used by Sato et al.,^46^ the latter was introduced in response to studies suggesting that HFIP might induce pre-aggregation.^62,73,74^ Both protocols yielded overlapping results in the kinetic analyses described below, with no clear difference between the two, indicating that HFIP pretreatment is not responsible for the observed variation. It should be noted that, at this stage, we only consider re-solubilization procedures without additional purification steps such as SEC, as these steps are not universally applied in the literature or protocols recommended by vendors, and, as will be shown later in this paper, they are not always necessary provided the appropriate re-solubilization procedures are used.

First, we used thioflavin T (ThT) assays to investigate the self-assembly kinetics of the Aβ(1-42) samples, as Aβ self-assembly kinetics are highly sensitive to the peptide’s composition and chemical environment, and so provide a quick and informative initial experiment to assess the quality and variability of preparations. ThT assays were carried out in polystyrene plates treated with a PEG-like low-binding surface, in a 20 mM sodium phosphate buffer (pH 8.0) containing 200 μM ethylenediaminetetraacetic acid (EDTA), 1 mM NaN_3_, and 20 μM ThT; in-house recombinant preparations have previously been shown to yield reproducible kinetics under these conditions.^15,39,75^ Peptide was prepared using either of the aforementioned protocols, with or without HFIP pretreatment, and self-assembly was initiated by rapid dilution into the fibrillization buffer to a final concentration of 1–10 μM peptide. As described in Section 3.3, since the peptide was dissolved in 10 mM NaOH prior to this addition, the fibrillization buffer was pre-adjusted to account for the addition of 10 mM NaOH alongside the Aβ(1-42). The success of this strategy was confirmed empirically.

**Figure 2:**
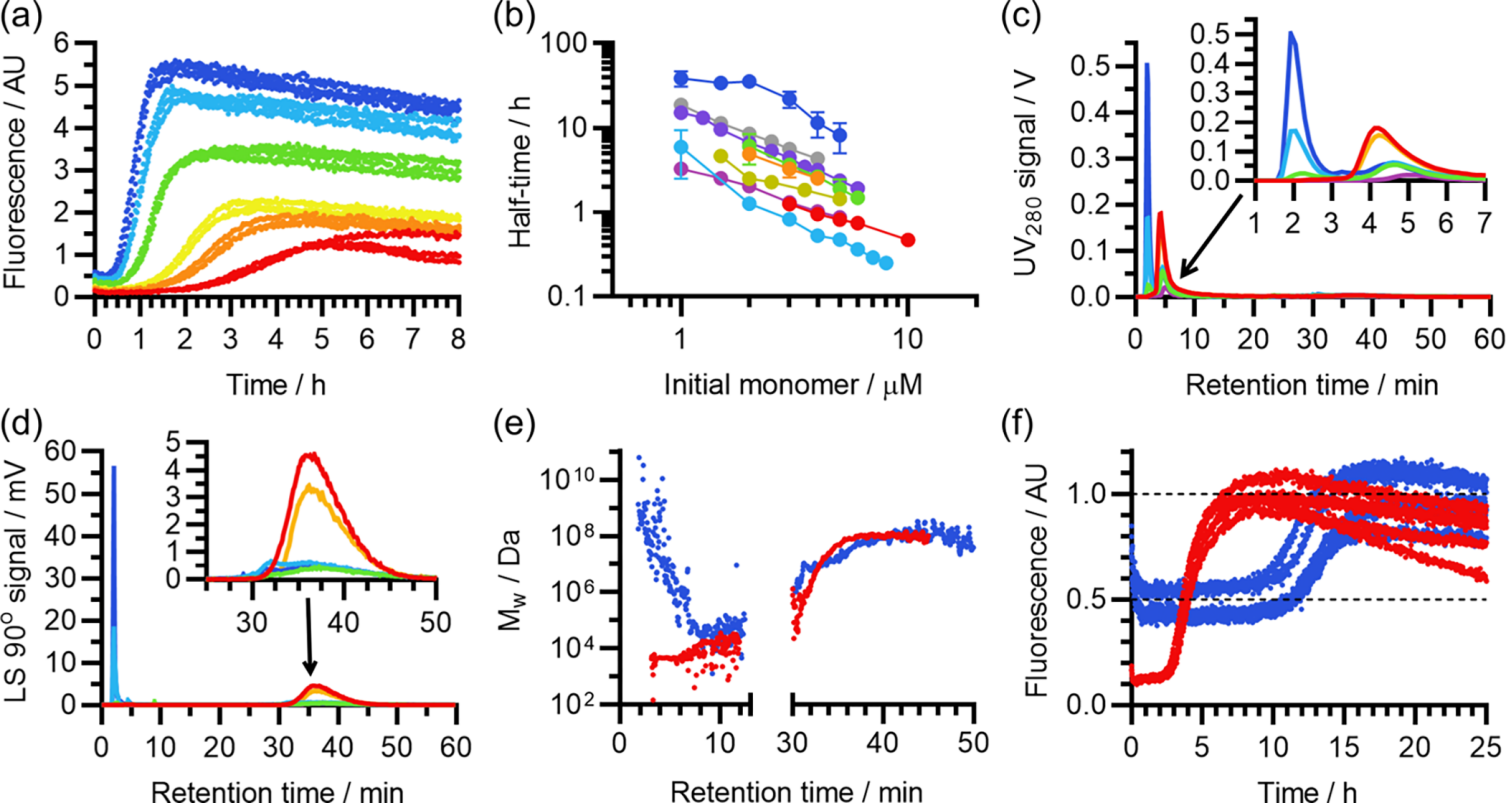
Incomplete monomerization of Aβ(1-42) re-solubilized in 10 mM NaOH. (a) Representative fibrillization kinetics of Aβ(1-42) re-solubilized in 10 mM NaOH without HFIP pretreatment (sample ID 10-30-N-4), viewed by ThT assay. The color scheme encodes the Aβ(1-42) concentration: red, 1 μM; orange, 1.5 μM; yellow, 2 μM; green, 3 μM; cyan, 4 μM; blue, 5 μM. Three replicate curves are shown for each Aβ(1-42) concentration. (b) Comparison of the concentration-dependent fibrillization half-times of nine different Aβ(1-42) samples solubilized in 10 mM NaOH. The color scheme indicates separate solubilizations, with (red/orange/yellow/green) or without (blue/indigo/violet/gray) HFIP pretreatment. Error bars indicate the standard deviation of replicate experiments with the same peptide sample. See Table S1 for the correspondence between the color scheme and sample ID. (c) Blank-subtracted UV_280_ elugrams from AF4-MALS analyses of Aβ(1-42) samples prepared in 10 mM NaOH without HFIP pretreatment, with each curve corresponding to the results of a single experiment. For clarity, data were selected from a broader body of experiments (6/13) to represent the full range of results. The sample ID and pH after re-solubilization were: red, 10-30-N-5, pH 10.0; amber, 10-30-N-6, pH 10.0; green/cyan/blue, 10-30-N-7, pH 7.4; violet, 10-30-N-8, pH 7.0. Note that the green, cyan, and blue curves are replicates conducted with the same re-solubilized peptide sample, demonstrating the variable recovery in peak 1; the blue curve most likely represents a full recovery. (d) The corresponding AF4-MALS elugrams showing light scattering signal at 90° (LS 90°), with the same color scheme as (c). (e) *M*_w_ elugrams corresponding to the red and blue curves in (c) and (d), with the same coloring. For clarity, only these two datasets are shown in this panel. (f) Representative ThT self-assembly kinetics obtained with the same Aβ(1-42) samples as the red and blue curves in (c) and (d). ThT experiments were conducted at different times and with different gain settings; to allow comparison, each ThT curve has been normalized relative to the maximum mean fluorescence of its own set of replicates.

As shown by the representative example in Figure 2a, the self-assembly curves were mostly sigmoidal, but had a nonzero initial fluorescence, indicating that some aggregation had occurred prior to the start of the experiment. In addition, as shown in Figure 2b, there was a high degree of variation between the self-assembly half-times of peptide samples solubilized on separate occasions, and the relationship between the initial Aβ(1-42) concentration and the self-assembly half-time was also weaker than expected, and highly variable. The concentration dependence is significant as it reflects the mechanism and stoichiometry of the underlying self-assembly processes, and it can be quantified by the scaling exponent γ, which is the slope when the half-time is plotted against the Aβ(1-42) concentration on double-logarithmic axes. For Aβ(1-42) concentrations in the 1–5 μM range, the average fitted value was γ = −1.04, with a standard deviation of 0.24 across 9 peptide samples, compared to a value of γ ≈ −1.3 previously determined for high-quality in-house recombinant preparations.^15,75^ The majority of kinetic variation was observed between Aβ(1-42) samples that were solubilized on separate occasions, rather than repeat experiments with the same sample, and there was no clear dependence on the batch, experimenter, or whether samples were pre-treated with HFIP. Thus, it appears that poor control of one or more experimental variables during solubilization caused incomplete monomerization or pre-aggregation, and this was responsible for the irreproducible self-assembly kinetics.

**Figure 3:**
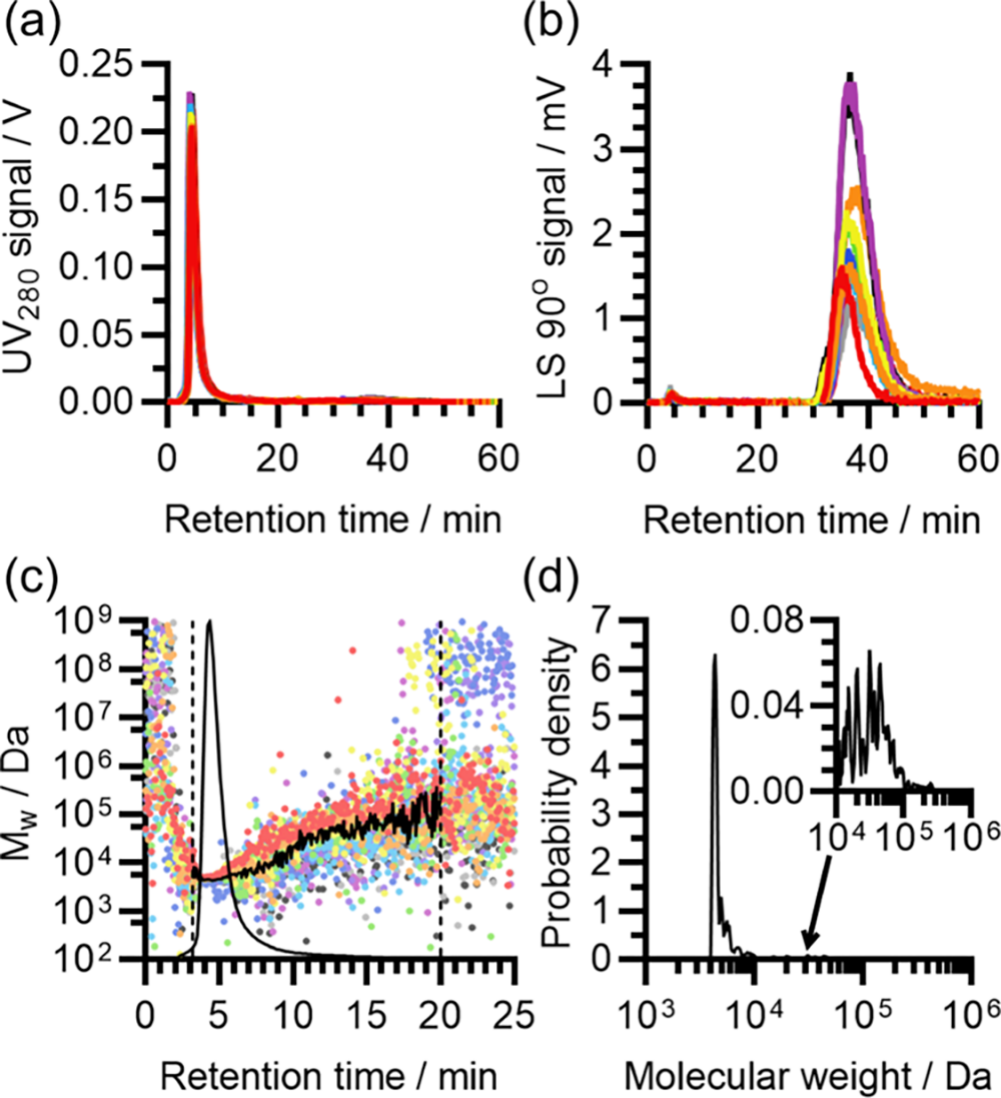
AF4-MALS analysis of Aβ(1-42) samples previously solubilized with 50 mM NaOH. (a) Blank-subtracted UV_280_ elugrams from the AF4-MALS analyses, with each curve corresponding to the results of a single experiment (*n* = 12). (b) Corresponding LS 90° elugrams, with the same color scheme. (c) Overlay of *M*_w_ elugrams obtained by analysis of 10 concordant experiments in (a) and (b), with the same color scheme. Two experiments were excluded due to a slight difference in the volume of the detector tubing, which caused a small peak shift that affected averaging and collation of results. Calculated *M*_w_ values are represented as points on a logarithmic axis, and the geometric average *M*_w_ for material eluting from 3.2 to 20 min is represented by the noisy black line. The average UV_280_ signal has been included for reference (smooth black line, linear scale, relative units), and the dashed vertical lines represent the boundaries of the region of interest (ROI) used to construct molecular weight distributions. The minor ticks on the *M*_w_ axis represent multiples of 5, i.e., 5 × 10^2^, 5 × 10^3^, 5 × 10^4^ Da, and so on. (d) Logarithmically corrected molecular weight distribution, showing the frequency density of eluted material with estimated molecular weight *M*_w_, by mass. Derived from the analysis of the ROI in (c). For all panels, see Table S2 for the correspondence between the color scheme, experiment, and sample ID.

To test this hypothesis, Aβ(1-42) samples prepared without HFIP treatment were analyzed by AF4-MALS, in a 1 mM NaOH mobile phase. Figure 2c shows the UV absorbance at 280 nm (UV_280_) from selected AF4-MALS runs, chosen to represent the full range of results, while Figure 2d shows the corresponding light scattering signal at 90° (LS 90°), and Figure 2e shows the estimated average molecular weight (*M*_w_) of the eluting material, based on analysis of the UV_280_ and MALS signals. Note that, due to low UV_280_ at the start of elution, the *M*_w_ estimates for the red and blue datasets in Figure 2e have been truncated at 3.3 and 1.8 min, respectively; this simply reflects the fact that *M*_w_ estimates before this time were unreliable, very noisy, and not suitable for further analysis. Comparison of the elugrams revealed three main peaks, whose amplitude varied between AF4 runs:

1. Peak 1 lasted from 1.5 to 3.3 min, reached a maximum around 2.0 min, and often had a strong UV_280_ and MALS signal. The timing of this peak identifies it as the system peak, which is routinely observed in AF4 runs and contains unretained material eluting in a single channel volume. For the experimental setup used in this study, the system peak would be expected to contain high molecular weight (HMW) material that was sterically occluded by contacts with the channel membrane, as supported by the strong MALS signal and the high *M*_w_ estimate (≳10^8^ Da) for the blue dataset in Figure 2e. Thus, peak 1 could contain large amyloid fibrils, clumps of amyloid fibrils, amorphous aggregates, and other HMW contaminants.
2. Peak 2 lasted from 3.3 to 10.0 min, reached a maximum from 4.2 to 5.0 min, and had a strong UV_280_ signal and a weak MALS signal. The timing of this peak indicates that it consisted of low molecular weight (LMW) material eluting in normal mode, and this assessment is supported by the weak MALS signal. In some cases, such as the blue dataset in Figure 3c–e, the tail of peak 1 overlapped with peak 2, making *M*_w_ determination of the latter unreliable. In other cases, such as the red dataset in the same panels, the two peaks were distinct, and the *M*_w_ was estimated at ∼4500 kDa at 4.2 min, rising gradually afterward. Thus, peak 2 probably contained monomer with a tail of oligomer, and the lack of a second peak indicates that the oligomers had a relatively broad size distribution.
3. Peak 3 lasted from 30.0 to 60.0 min, was relatively broad, and had a weak UV_280_ signal and a strong MALS signal. The timing and strong MALS signal of this peak indicate that it consisted of large material that had reversibly adhered to the channel membrane, and later detached once the cross-flow was relaxed. Material likely to elute in peak 3 would include amyloid fibrils and other large aggregates but also nonprotein contaminants such as dust and microorganisms.

On average, out of 20 μg of peptide injected, peak 1 (1.5–3.3 min) contained a nominal 3.99 ± 5.92 μg of material, peak 2 (3.3–30.0 min) contained 11.73 ± 5.70 μg, and peak 3 (30.0–60.0 min) contained 2.11 ± 0.95 μg, where the error margins represent the standard deviation in each case. Note that the high standard deviation of peak 1 reflects a very broad, non-Gaussian distribution of mass estimates. In total, an estimated 17.82 ± 6.68 μg of material eluted in the course of the average AF4 run. This is not significantly different from the expected recovery of 20 μg, with a *t*-test between the two giving *p* = 0.63, and discrepancies in the estimated recovery of individual samples are likely to result from the conflicting effects of turbidity and partial adhesion of HMW constituents to the membrane. Therefore, given a true injected mass of 20 μg, the above recovery estimates indicate that around 20 ± 30% of sample eluted in peak 1, 59 ± 29% in peak 2, and 11 ± 5% in peak 3. This indicates that, typically, only around half of the sample was monomer or small oligomer, and the rest was in a highly aggregated state, with the extent of aggregation varying significantly between replicate runs. These results support the hypothesis that incomplete and inconsistent monomerization was responsible for the variable self-assembly kinetics.

A clue to the cause of incomplete monomerization was provided by the pH of the re-solubilized Aβ samples. First, it was observed that the pH of the 10 mM NaOH used to re-solubilize the samples dropped significantly during dissolution, from an initial value of 12.0 prior to injection into the vials, to a final value of 6.5–10.5 after extraction. The concentration of Aβ(1-42) was too small to explain such a change, indicating that variable concentrations of residual acids that were already in the vials had partly neutralized the base. Since Garai and Frieden^72^ showed that the threshold for eliminating aggregation is between pH 11.0 and pH 12.0, all of these samples were within the aggregating range. Second, a correspondence was observed between the pH of re-solubilized Aβ samples, their composition revealed by AF4-MALS, and their performance in ThT assays. As can be seen in Figure 2c–e, preparations with a higher pH (≳10.0) after re-solubilization had a larger quantity of material eluting in peak 2 (18.30 ± 1.17 μg), which corresponds to monomer and small oligomer, whereas preparations with a more neutral pH (≲8.0) had a much smaller quantity of material in this peak (7.62 ± 2.20 μg). This difference was found to be significant by a two-sample *t*-test (*p* = 8.2 × 10^–7^). Conversely, as can be seen from the same figure, high-pH samples had a relatively low recovery in peak 1 (0.25 ± 0.09 μg), whereas more neutral samples had a high and very variable recovery in peak 1 (6.32 ± 6.62 μg). This difference was less significant (*p* = 0.069), but the reduced significance probably reflects the very high standard deviation of the low pH samples, which is itself notable. This variation is probably due to difficulties with injection or recovery rather than true sample variation, as samples with pH < 8.0 were highly viscous, and difficult to mix and inject. Alternatively, highly aggregated samples can sometimes adhere irreversibly to the membrane, in which case the sample would not be seen. A significant difference was not observed for peak 3, suggesting that the material that adhered reversibly to the membrane was not involved in self-assembly. In line with the observed variation in sample composition, there was substantial variation in the self-assembly kinetics. As shown in Figure 2f, the self-assembly kinetics of Aβ(1-42) preparations that had final pH > 10.0 were relatively close to what would be expected for nucleated polymerization without pre-aggregation, with a well-defined growth phase and a comparatively low initial ThT fluorescence, whereas preparations with final pH < 8.0 showed clear signs of off-pathway pre-aggregation, with a high initial fluorescence and a strongly delayed growth phase. Thus, analysis of sample pH suggested that variable neutralization of the solvent was responsible for pre-aggregation, and differences in pre-aggregated species between peptide samples explained the inconsistency of the kinetics. In addition, the fact that the most severely affected samples had retarded self-assembly kinetics suggested that the pre-formed aggregates were mostly off-pathway.

### 2.3 Improved Solubilization Protocol Using 50 mM NaOH Yields Highly Monomeric Preparations

The data presented in Section 2.2 lead to two separate conclusions regarding the efficacy of dilute base treatments for re-solubilizing Aβ. First, detectable aggregation occurs in the pH 10.0–10.5 range, in agreement with previous work showing that the threshold for preventing aggregation is somewhere in the pH 11.0–12.0 range.^72^ Thus, protocols that yield samples with a pH up to 10.5, and possibly as high as 12.0, risk pre-aggregation and ill-defined self-assembly behavior as a result. Second, dilute base solvents are highly susceptible to neutralization by residual acids in peptide samples, which exacerbates the above issue. Since many studies use a basic solvent with a pH of 10.6–12.0 prior to neutralization,^41,46,48,53,56,57^ and most do not report checking the pH, partial neutralization may thus be a widespread issue. Therefore, to improve both the quality and reliability of preparations, it is necessary to ensure that the solvent is sufficiently concentrated that the pH will be outside the aggregating range (ideally ⩾ 12.0), and that pH neutralization upon dissolution does not move the pH into the aggregating range.

While one option is to accept incomplete dissolution and then purify re-solubilized samples by SEC, there are several issues with this approach. First, additional rounds of SEC result in a significant loss of peptide and dilution of the eluted sample, compounding the issues caused by incomplete solubilization and limiting the peptide concentrations that can be used down the line. This negates the main advantages of commercial preparations, which are already supplied at a high level of purity, and in a lyophilized form that increases the flexibility of subsequent experiments. Second, to use Aβ(1-42) at even moderate concentrations (e.g., 1–6 μM) it is typically necessary to carry out SEC in the buffer that will be used for subsequent experiments. This is inconvenient, limiting the lifespan of the purified samples to a few minutes, and risks further pre-aggregation during elution and sample collection. Third, this approach also fails to address the suboptimal yields and high failure rate of existing base treatments, since SEC cannot recover Aβ(1-42) that has already aggregated; in contrast, even if it were still found to be necessary to perform SEC after solubilization, SEC of properly solubilized samples would be expected to achieve much higher yields. For these reasons, the decision was made to address the issue of partial neutralization at the point of dissolution, before attempting any additional purification steps.

The simplest means to achieve a higher and more reliable pH is to increase the concentration of NaOH. This ensures there is an excess of a strong base, preventing the formation of a buffered solution with any residual acids in the vials. In addition, the greater the concentration of base, the smaller the proportional variation in the concentration that will be neutralized, resulting in a more consistent pH. Therefore, a new protocol was proposed in which Aβ(1-42) was directly dissolved in 50 mM NaOH by sonication for 5 min, rapidly frozen in liquid N_2_, and then stored at −80 °C. The sonication time was reduced as a precaution to limit the exposure of the peptide to high pH while in the liquid phase. In most cases (92%), peptide samples solubilized in this manner had a final pH above 12.0, and the final pH was typically 12.5, which is close to the expected value for 50 mM NaOH. In a small number of cases (8%), without clear batch dependence, the pH was lower than 12.0, and near-neutral pH values were occasionally observed, indicating that a limited number of vials still contained enough residual acid to neutralize the solvent; in these cases, as with 10 mM NaOH samples that reached a similar pH, kinetics were slow or absent. However, neutralization of the 50 mM NaOH occurred with a much lower frequency than the 10 mM NaOH, and could easily be identified by taking pH measurements. In addition, as expected, the un-neutralized majority of samples had a much higher and more consistent pH than those prepared in 10 mM NaOH, with 92% having a pH outside of the suggested aggregating range.^72^ Therefore, a concentrated base leads to a marked reduction in the rate of preparation failure and results in preparations with a much higher and more consistent pH.

As shown in Figure 3a,b, initial AF4-MALS investigation, using the same method that was applied to samples prepared in 10 mM NaOH (Section 2.2), revealed that the high-pH samples were highly consistent. On average, 0.31 ± 0.17 μg of material eluted in the peak 1 region (1.5–3.3 min), 19.59 ± 0.90 μg in the peak 2 region (3.3–30.0 min), and a nominal 0.72 ± 0.32 μg in the peak 3 region (>30 min). In these results, peak 1 manifested as a shoulder at the start of peak 2, due to the close proximity and size disparity between the two, whereas peak 3 remained distinct. Compared to Aβ(1-42) prepared in 10 mM NaOH, there was a noticeable reduction in the variance of the quantity of material eluting in steric mode (1.5–3.3 min), a significant increase in the quantity of material eluting in normal mode (3.3–30.0 min; *p* = 3.2 × 10^–4^), and a significant reduction in the quantity of material eluting after cross-flow (30.0–60.0 min, *p* = 2.4 × 10^–4^). It is worth noting that the total recovery across all three regions of interest (20.62 ± 1.19 μg) was slightly larger than the expected value of 20 μg, although still within error, whereas the recovery from the first two regions of interest (1.5–30.0 min) was very close to this value (19.90 ± 0.99 μg). This suggests that close to 100% of peptide may have eluted during cross-flow, and the material eluting after cross-flow may instead have been other residual contaminants, such as dust; this assessment is supported by the analyses presented later in this paper.

The abundance of AF4-MALS data enabled a detailed analysis of the molecular weight distribution of the Aβ(1-42) that had been re-solubilized using this new protocol. Figure 3c shows an overlay of the *M*_w_ elugrams of 10 closely concordant experiments, based on analysis of the UV_280_ and MALS signals, and also shows the geometric mean of those elugrams from 3.3 to 20.0 min. Due to a difference in the size of the detector tubing, two experiments had a mild peak shift and were excluded from this analysis. In the region of interest (ROI) from 3.3 to 20.0 min, the *M*_w_ initially dropped due to declining quantities of sterically eluting material, remained level at ∼4500 Da from 4.0 to 5.0 min, and then gradually increased after that point due to the co-elution of small oligomers. In the geometric average *M*_w_ elugram, 88% of sample eluted in fractions with *M*_w_ closer to the monomer, and 12% eluted in fractions suggestive of dimer or larger. This probably somewhat underestimates the monomer content, as: (i) the material eluting before 3.3 min, which was excluded from the analysis, was probably monomer contaminated with trace amounts of sterically eluting material; (ii) the averaging process did not fully eliminate the noise from the *M*_w_ elugram, which will have exaggerated the oligomer content; and (iii) samples with *M*_w_ closer to dimer or larger may still have been predominantly monomer, with small quantities of particularly large species biasing the results. Nonetheless, although this analysis suggests that the LMW fraction consisted predominantly of monomer, it is clear that small quantities of oligomeric species were present.

The *M*_w_ elugrams used to obtain the geometric average were highly concordant, and most variation between them appeared to be either noise or baselining error. Therefore, taking advantage of the improvement in signal-to-noise ratio, the average elugram was combined with the UV_280_ signal to construct an approximate *M*_w_ distribution for the sample eluting from 3.3 to 20.0 min, as shown in Figure 3d. The *M*_w_ distribution represents the frequency density of Aβ(1-42) mass eluting in fractions with different *M*_w_ values at the time of detection, and includes a correction to preserve the peak area while using a logarithmic *M*_w_ axis. The corresponding cumulative elugram is shown in Figure S1. Consistent with the lack of additional peaks in the UV_280_ elugrams, the molecular weight distribution has a single major peak at ∼4500 Da corresponding to the Aβ(1-42) monomer. The tail to the right-hand-side of the peak reflects the presence of fractions containing a mixture of monomer and oligomers, and the small peak from 10^4^ to 10^5^ Da may reflect the presence of fractions containing mainly 2–20 mers, or alternatively a mixture of retarded monomer and larger species. The finer peaks in that region are the result of noise, and should not be interpreted.

Altogether, the AF4-MALS data indicate that high-pH treatment causes almost complete dissolution of the Aβ(1-42), and that the dissolved peptide is predominantly monomeric, with small but significant quantities of oligomers detected during elution. It is unclear whether these oligomers existed in the initial preparations, or were induced by pH changes occurring in the channel since injection of the sample into the 1 mM NaOH mobile phase would have brought the peptide into conditions where limited aggregation could occur on the timescale of the AF4 run.^72^ If the oligomers were present in the preparations themselves, their formation was probably induced by the high Aβ(1-42) concentrations, as Aβ(1-42) monomers would otherwise be expected to repel one another strongly at pH 12.5. Small oligomers are inevitable in all Aβ(1-42) preparations, due to the peptide’s high aggregation propensity and the requirement to prepare samples at high concentrations. There do not appear to be any equivalent analyses of the oligomer content of Aβ(1-42) preparations in the literature, as most protocols use more qualitative approaches to check for oligomers, such as negative-stain electron microscopy (NS-EM) and gel electrophoresis. In addition, NS-EM struggles to identify aggregates with diameter < 5 nm, and electrophoretic techniques are unlikely to detect oligomers that have a very broad size distribution and consequently a low individual abundance. Nonetheless, since the conditions used here are more denaturing than those used to monomerize Aβ(1-42) in most other studies, and methods such as filtration^41,47,54,58,60,61,64,68^ and centrifugation^19,56,76^ that are commonly used to “monomerize” the protein would be unable to remove most of the species observed here, it seems likely that other preparations would give similar results if subjected to the same analysis. Furthermore, it is shown in Section 2.5 that isolation of the monomer peak by SEC does not affect the self-assembly kinetics, despite purportedly increasing the monomer content of the purified sample. The simplest interpretation of this result is that any oligomers formed at high pH and peptide concentration rapidly equilibrate with the monomer upon dilution into the fibrillization buffer so that the free monomer content of SEC-treated and untreated samples are ultimately the same. This argument does not negate the possible role of SEC in removing persistent oligomers or on-pathway species such as fibril seeds, which is evaluated in the next section.

### 2.4 Aβ(1-42) Samples Solubilized in 50 mM NaOH Exhibit Highly Reproducible, Unseeded Self-Assembly Kinetics

**Figure 4:**
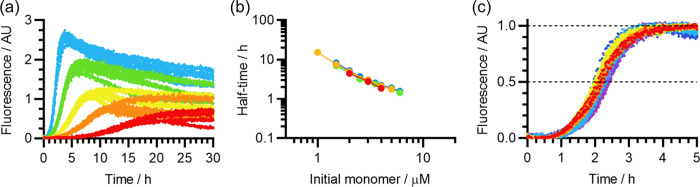
Self-assembly kinetics of Aβ(1-42) solubilized with 50 mM NaOH prior to use in ThT assays. Self-assembly was induced by dilution of the high-pH Aβ(1-42) sample into a pH-corrected 20 mM sodium phosphate buffer (pH 8) containing 200 μM EDTA, 1 mM NaN_3_, and 20 μM ThT, at 37 °C. (a) Fibrillization kinetics of a single representative Aβ(1-42) sample solubilized in 50 mM NaOH (sample ID 50-5-N-11), viewed by ThT assay. Color scheme encodes Aβ(1-42) concentration: red, 1 μM; orange, 1.5 μM; yellow, 2 μM; green, 3 μM; cyan, 4 μM. Five replicates are shown for each Aβ(1-42) concentration. (b) Comparison of the concentration-dependent fibrillization half-times of four samples of peptide individually solubilized in 50 mM NaOH. Color scheme indicates different peptide samples, with ID: red, 50-5-N-10; amber, 50-5-N-11; green, 50-5-N-12; blue, 50-5-N-13. Each data point is the mean across 4–20 fibrillization curves, from 1 to 5 experiments with 3–8 replicate wells. For all datasets, the standard deviation across replicates is too small to represent in this panel. A total of 303 self-assembly curves are represented by the averages in this panel. (c) Overlay of the ThT self-assembly kinetics of 4 μM Aβ(1-42) from eight samples of peptide individually solubilized in 50 mM NaOH, normalized relative to their maximum fluorescence to correct for varying gain between experiments. The color scheme indicates different peptide samples. For each sample, 3–25 self-assembly curves are shown, from 1 to 5 experiments with 3–5 replicate wells. A total of 88 self-assembly curves are shown in this panel. All data points are shown. See Table S2 for the correspondence between the color scheme, experiment, and sample ID.

To determine whether Aβ(1-42) samples prepared in 50 mM NaOH exhibited controlled, unseeded fibrillization, ThT assays were carried out under the same conditions previously used for 10 mM NaOH Aβ(1-42). As before, the fibrillization buffer was pre-adjusted so that it would reach the correct pH when the Aβ(1-42) and accompanying 50 mM NaOH were added (Section 3.3); the success of this strategy was confirmed by pH measurements, and further adjustments were never needed after adding the peptide. The results of these ThT assays are shown in Figure 4. Consistent with a predominantly monomeric composition and lack of large populations of HMW species, Aβ(1-42) samples prepared in 50 mM NaOH had a low initial ThT fluorescence (mean 2.2% of final fluorescence at 4 μM peptide), and exhibited classically unseeded sigmoidal fibrillization kinetics with a distinct lag phase. Moreover, unlike Aβ(1-42) prepared in 10 mM NaOH, samples prepared in 50 mM NaOH produced highly consistent self-assembly kinetics, with the fibrillization half-time having a proportional standard deviation of 4.9% at 4 μM Aβ(1-42), and the concentration-dependences from different samples aligning almost exactly. The small remaining variations in the half-time did not correlate with the initial ThT fluorescence, indicating that they were not due to the presence of seed (Figure S2). Since the main difference between samples prepared in 10 mM NaOH and those prepared in 50 mM NaOH was the increase in monomer content and removal of HMW material from the latter, these results confirm that pre-aggregation was responsible for the inconsistent kinetics of Aβ(1-42) solubilized in 10 mM NaOH.

### 2.5 NS-EM and SEC Confirm the Lack of Fibril Seeds in Aβ(1-42) Solubilized in 50 mM NaOH

**Figure 5:**
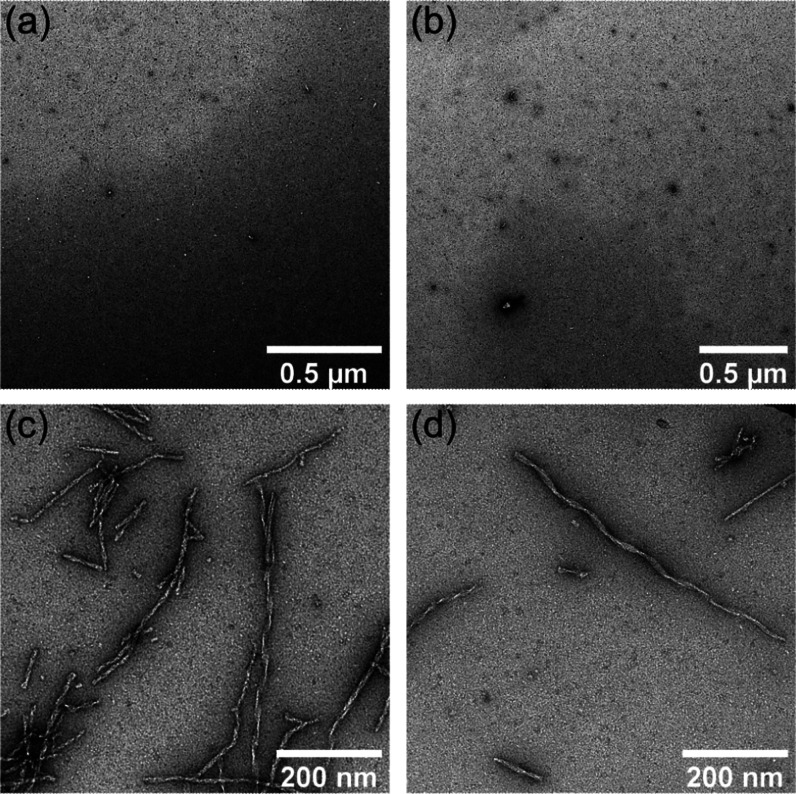
NS-EM of 4 μM Aβ(1-42) solubilized with 50 mM NaOH and then diluted into pH-corrected fibrillization buffer containing 20 mM sodium phosphate (pH 8), 200 μM EDTA, 1 mM NaN_3_, and 20 μM ThT. (a, b) Representative images of Aβ(1-42) samples immediately after the dilution. (c, d) Early growth phase samples from the same experiment, after incubation for ∼1 h in a 96-well microplate at 37 °C.

**Figure 6:**
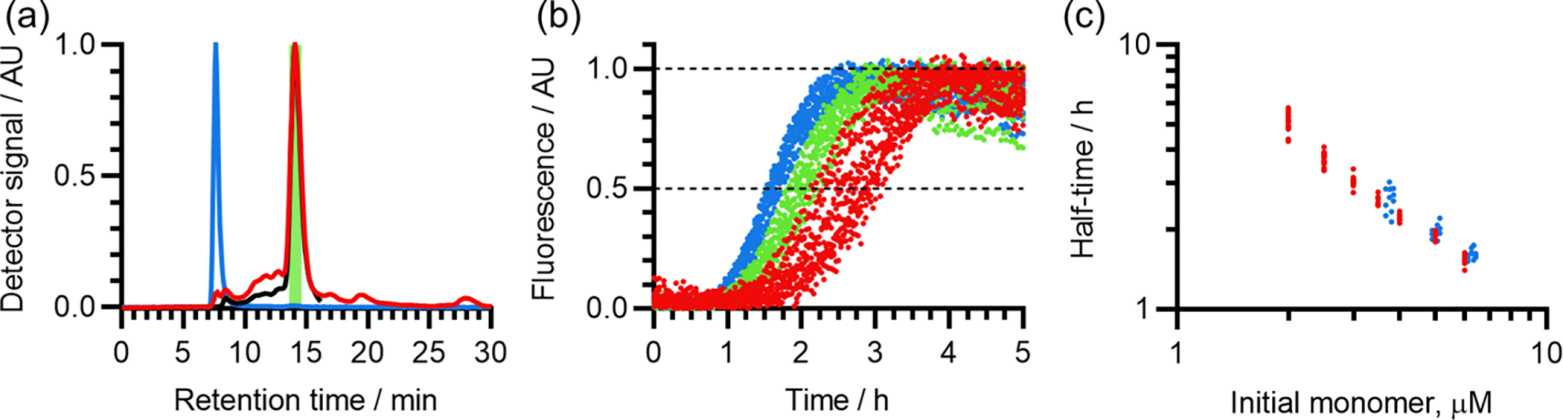
SEC purification does not affect the self-assembly kinetics of high-pH Aβ(1-42) preparations. Aβ(1-42) that had been solubilized in 50 mM NaOH was purified using a Superdex 75 column and then used in ThT assays at 37 °C. The buffer used for elution and subsequent ThT assays was sodium phosphate (pH 8) containing 200 μM EDTA and 1 mM NaN_3_, and the sample was eluted at 1 mL/min. (a) Detector outputs from a typical SEC-MALS run of Aβ(1-42) prepared in 50 mM NaOH. Color scheme: black, RI; red, UV_280_; blue, LS 90°. The region colored green indicates the monomer-containing fraction (13.6–14.6 min), which was collected for ThT assays. (b) Normalized ThT curves of purified Aβ(1-42). Color scheme indicates Aβ(1-42) concentration as a percentage of the eluent concentration: red, 60% (∼3.8 μM); green, 80% (∼5.0 μM); blue, 100% (∼6.3 μM). (c) Similarity between the fibrillization half-times of unpurified (red) and SEC-purified (blue) Aβ(1-42), both from the same re-solubilized peptide sample (ID 50-5-N-13).

The very low initial ThT fluorescence and high level of kinetic reproducibility indicated that the self-assembly kinetics of Aβ(1-42) prepared in 50 mM NaOH was not confounded by seeding or off-pathway aggregation, which would be expected to vary between preparations. In agreement with this, as shown in Figure 5, negative-stain electron microscopy (NS-EM) did not reveal any fibrillar species present in 4 μM Aβ(1-42) samples immediately after dilution into pre-adjusted fibrillization buffer, whereas fibrils were observed after incubation for ∼1 h under fibrillization conditions, equivalent to the early growth phase in ThT assays. To perform a further test for the presence of seeds, and determine whether the detected oligomers affected the self-assembly kinetics, Aβ(1-42) samples were purified by SEC, and their self-assembly kinetics were compared to those of unpurified samples. In summary, following the protocol used by Hellstrand et al.,^39^ 50 μL of Aβ(1-42) was loaded onto a Superdex 75 column equilibrated with a 20 mM sodium phosphate (pH 8) mobile phase, with 200 μM EDTA and 1 mM NaN_3_. The fraction eluting from 13.6 to 14.6 min (at 1 mL/min) was collected on ice, diluted variably (60, 80, or 100%, i.e., undiluted) in the elution buffer to give a range of concentrations, supplemented with ThT from a concentrated stock, and used in ThT assays. This purification procedure was repeated several times with reproducible results, and a representative elugram is shown in Figure 6a. The UV absorbance and RI elugrams are very similar to those previously reported for in-house recombinant peptide purified according to the same protocol,^39^ and the peak at ∼14 min has already been identified as the monomer. MALS analysis of the ROI from 13.6 to 14.6 min was challenging, as the very weak scattering signal meant that the *M*_w_ estimates were particularly sensitive to baseline subtraction errors. Nonetheless, the average *M*_w_ value of this ROI across three concordant replicates was 6460 ± 712 Da, where the error margins represent one standard deviation, consistent with the peak containing mostly monomer with a small but significant amount of contaminating oligomer. It is worth noting that, despite claims in the literature,^39,65,77^ this SEC protocol does not yield particularly monomeric Aβ(1-42) solutions. As discussed previously, some oligomeric species are inevitable in any Aβ(1-42) preparation, and, in this case, the purification protocol was either unable to completely separate the pre-formed oligomers or actively encouraged re-formation of oligomers during elution since purification was carried out under aggregation conditions.

The total mass of Aβ(1-42) in the fraction collected from 13.6 to 14.6 min was 28.4 ± 7.13 μg, equivalent to 56.8% of the injected mass at a concentration of 6.29 μM, approximately 35× more dilute than the concentration at which the sample was injected. For each purification, the Aβ(1-42) was then diluted in the same buffer to 60, 80, or 100% (undiluted) of that concentration, supplemented with ThT, and used in a ThT assay. Exact concentrations of Aβ(1-42) in individual experiments were then calculated retrospectively for use in further analyses. An overlay of all concordant ThT self-assembly curves is shown in Figure 6b, in which the kinetics can be seen to have broadly the same rate and characteristics as peptide that had not been purified by SEC. Because the Aβ(1-42) eluted at a variable concentration, an exact overlay of the self-assembly kinetics of SEC-treated samples with those of untreated samples was not possible. However, the relative rates can still be compared by overlaying the concentration-dependences of the fibrillization half-times. As shown in Figure 6c, the fibrillization half-times of the SEC-treated and untreated Aβ(1-42) samples overlay almost exactly. While it is possible that the SEC-treated samples may be slightly slower, the difference between the two is very minor and well within experimental variation. Even a small quantity of seed would be expected to strongly affect the fibrillization rate; however, SEC has little if any effect on the half-time, indicating that the untreated samples did not contain a significant level of seed. If an effect does exist, it is very small and more consistent with the removal of very low quantities of heterogeneous contaminants such as dust or microorganisms, which may weakly stimulate heterogeneous primary nucleation. This conclusion was further supported by the removal of the HMW material by ultracentrifugation, which also had a negligible impact on the self-assembly kinetics (Figure S4). Therefore, for samples solubilized according to our protocol, SEC results in significant loss and dilution of the peptide, places considerable constraints on its use in subsequent experiments, fails to identify fibril seed in the untreated samples, and does not significantly affect the self-assembly kinetics. As a result, we propose that proper solubilization largely eliminates the need for additional purification of lyophilized Aβ samples, provided rigorous purification procedures have previously been applied. Nonetheless, we continue to recommend the use of additional purification steps for samples that are supplied at a lower level of purity or contain residual solvents or counterions that affect the results, as well as in control experiments where it is important to exclude the possibility of contamination or seeding.

### 2.6 Aβ(1-42) Is Not Chemically Modified during Solubilization and Handling in 50 mM NaOH

**Figure 7:**
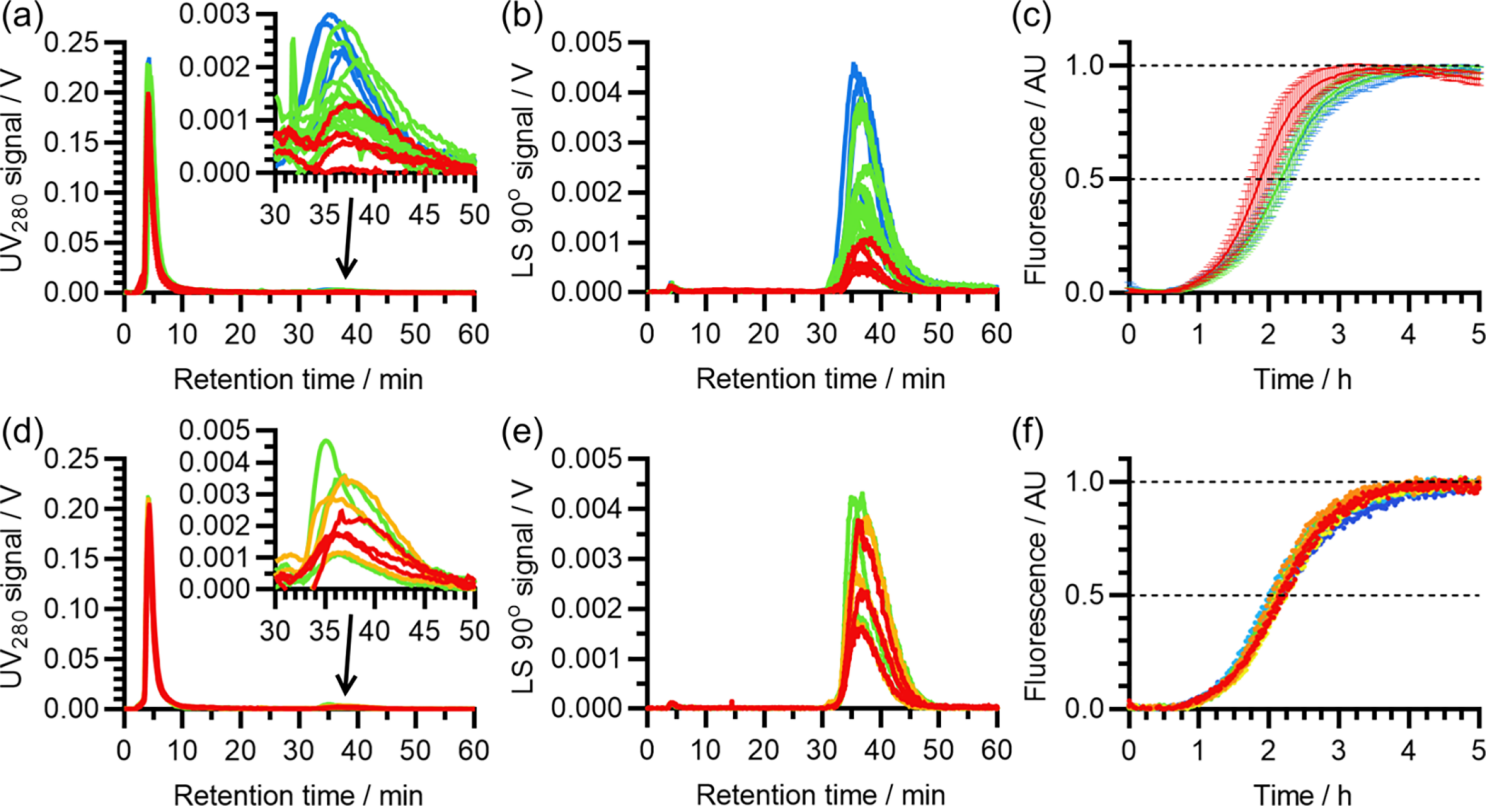
Effect of sonication at high pH and subsequent further exposure to high pH on the size distribution and self-assembly kinetics of Aβ(1-42) samples. (a–c) Effect of varying the sonication time during solubilization in 50 mM NaOH, with the color scheme reflecting this variation: red, 0 min (no sonication); green, 5 min; blue, 30 min. (a) The UV_280_ signal from AF4-MALS separation of the aforementioned samples. (b) Corresponding LS 90° signal. (c) Normalized ThT self-assembly kinetics of samples that were subsequently diluted into pre-adjusted fibrillization buffer, as described in the text. Error margins represent a single standard deviation. (d–f) Effect of subsequent incubation at 21 °C on the size distribution and self-assembly kinetics of Aβ(1-42) that had been prepared in 50 mM NaOH with 5 min sonication. In this way, samples were given additional exposure to high pH (∼12.5) for varying amounts of time. (d) UV_280_ signal from AF4-MALS separation of the aforementioned samples. The color scheme represents the incubation time: red, 10 min; amber, 140 min; green, 270 min. (e) Corresponding LS 90° signal, using the same color scheme as (d). (f) Normalized ThT self-assembly kinetics of samples that were subsequently diluted into pre-adjusted fibrillization buffer, as described in the text. The color scheme reflects the incubation time at 21 °C, but is different from (d) and (e): red, 15 min; orange, 60 min; yellow, 120 min; green, 300 min; cyan, 600 min; blue, 1200 min.

One of the primary concerns with the switch to a higher pH solubilization protocol was the risk of chemical modification. For this reason, the 50 mM NaOH solubilizations discussed above had all been carried out with 5 min sonication followed immediately by rapid freezing, as opposed to the 30 min sonication previously used for 10 mM NaOH solubilizations. Nonetheless, at this stage, experiments had not yet been performed to establish whether chemical modification posed a significant risk on these timescales; in addition, it was not yet clear whether a sonication step remained necessary when using a more concentrated solvent. Therefore, AF4-MALS experiments and ThT assays were used to compare the composition and self-assembly kinetics of Aβ(1-42) samples that had been solubilized in 50 mM NaOH with three different sonication times: 0 min, i.e., immediate rapid freezing without sonication; 5 min, i.e., the data acquired with the original 50 mM NaOH protocol; and 30 min, as in the previous 10 mM NaOH protocol. As shown in Figure 7a,b, there was no obvious difference between the AF4-MALS elugrams of samples that were sonicated for 0, 5, or 30 min. Similarly, ThT assays revealed a similar initial and final fluorescence for all three sets of samples (Figure S3). As shown in Figure 7c, nonsonicated samples did appear to self-assemble slightly faster, suggesting that some seeds may have been present in the initial mixture. However, there was no difference between the self-assembly kinetics of samples that were sonicated for 5 and 30 min, indicating that the seed was eliminated within the first 5 min of sonication. This supports our observation that there is no detectable seed in samples sonicated for 5 min, and further purification steps do not improve sample quality.

In addition, given the sensitivity of Aβ self-assembly to even small changes in primary sequence or length, the fact that there was only a small change in rate between 0 and 5 min of sonication, and no further change between 5 and 30 min, indicates that there was no significant chemical modification on this timescale. This conclusion was further corroborated by liquid chromatography–mass spectrometry (LC–MS), as there was no obvious difference between the mass spectra of samples that were sonicated for 5 and 30 min (Figures S5 and S6). Although one plausible modification, sidechain deamidation, involves too small a change in molecular weight to be detectable by our AF4-MALS and LC–MS analyses (+1 Da), given the importance of amide ladders in maintaining amyloid fibril structures,^78−80^ the existence of amide ladders in most Aβ(1-42) fibril structures (e.g., refs (81−85)), and the established sensitivity of Aβ(1-42) self-assembly kinetics to mutations that perform the inverse of this process,^86^ we believe that the lack of a significant change in the self-assembly kinetics makes it seem highly implausible that deamidation occurred on these timescales. Thus, we do not believe that our high-pH treatment results in significant chemical modification, and we propose that a brief sonication period of 5 min is necessary and sufficient to remove any pre-formed seed.

Although our data indicated that Aβ(1-42) was not chemically modified by spending short periods of time in 50 mM NaOH, it was useful to determine whether longer timescales or harsher treatments could result in modification. To test this, Aβ(1-42) aliquots from a single sample prepared by 5 min sonication in 50 mM NaOH were thawed and incubated at 21 °C, for times up to 20 h. As shown in Figure 7d–f, incubation at 21 °C did not significantly affect the AF4-MALS or ThT data, indicating that chemical modification and degradation were not significant on these timescales. This shows that, while it is still best practice to minimize the amount of time spent at high pH in the liquid phase, the peptide is relatively stable under these conditions and attempts to minimize this time should not be made at the expense of other experimental precautions.

### 2.7 Storage at −80 °C Ensures Long-Term Sample Stability

**Figure 8:**
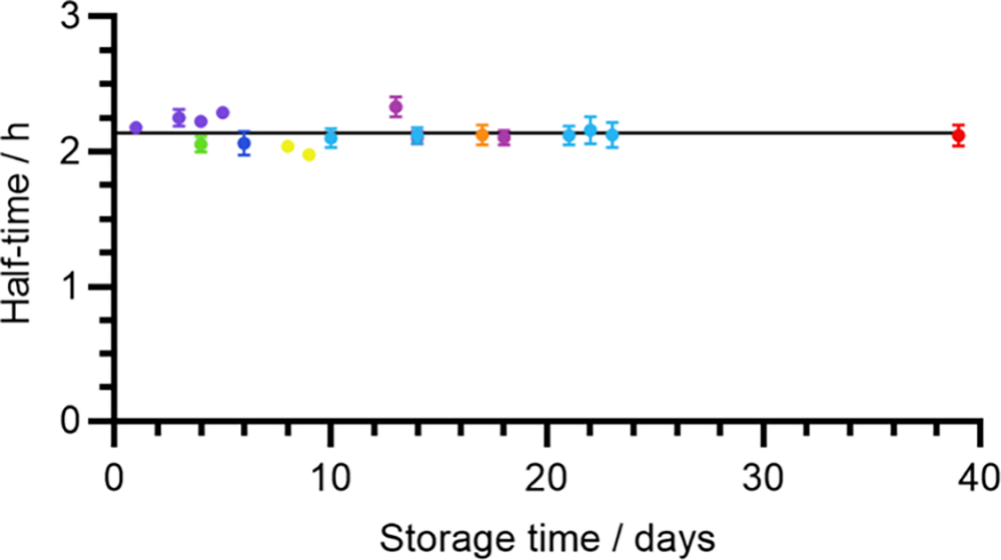
Effect of storage time on the fibrillization half-time of Aβ(1-42) samples solubilized by 5 min sonication on 50 mM NaOH. Each point corresponds to the average fibrillization half-time of a single ThT experiment; the error bars, which in some cases are too small to show, represent a single standard deviation. The color scheme indicates individual Aβ(1-42) preparations and corresponds to the experiments shown in Figure 4c. The fitted curve is an exponential decay of the form τ_half_ = τ_half_^′^*e*^–*k*_mod_*t*^, where τ_half_ is the self-assembly half-time, τ_half_^′^ is the half-time prior to the effects of storage, *k*_mod_ is the proportional rate of change of τ_half_ during storage, and *t* is the storage time. See text for values of the fitted parameters and Table S2 for the correspondence between the color scheme and sample ID.

It was also desirable to assess the long-term stability of Aβ(1-42) during storage. The Aβ(1-42) used in the experiments shown in Figure 4 had been kept at −80 °C for a range of storage times before use, from 1 to 39 days. Despite this, the data are highly consistent, indicating that the peptide was stable at −80 °C and did not undergo significant modification during storage. To more quantitatively assess this stability, the mean fibrillization half-time τ_half_ of each ThT experiment was plotted against the storage time of the corresponding aliquot. As shown in Figure 8, there is little to no relationship between the two. Fitting an exponential curve to these data gave a very slow decay in the self-assembly half-time over the duration of storage, with a rate constant *k*_mod_ = 6.53 × 10^–4^ day^–1^ and corresponding time constant τ_mod_ = 1530 days ≈ 4 years. To determine whether this fit suggested a significant level of degradation, the fitted *k*_mod_ value was then compared to a hypothetical value of *k*_mod_ = 0 using the extra-sum-of-squares *F*-test. This gave a *P*-value of 0.3011, too high to reject the null hypothesis that *k*_mod_ = 0, thus indicating that a significant level of degradation was not observed for this dataset. Therefore, Aβ(1-42) samples prepared with 5 min sonication in 50 mM NaOH are stable for well over 39 days at −80 °C. While we have not performed a systematic analysis on longer timescales, we note that this very slow decay suggests a long period of stability, perhaps lasting several years, and at the very least the tested period of 39 days is highly convenient for batch preparation and later experimentation.

### 2.8 Commercial Recombinant Aβ(1-42) Has an Identical Self-Assembly Pathway to In-House Recombinant Preparations

Our results show that sonication in 50 mM NaOH provides a reliable means to obtain highly monomeric Aβ(1-42) samples from a pre-purified commercial recombinant source. Peptide solubilized in this way is stable for at least 20 h in the liquid phase, can be stored for a matter of months or possibly years at −80 °C, is convenient for use in high-throughput assays, and exhibits very reliable self-assembly kinetics that are not complicated by detectable seeding, chemical modification, or the effects of contaminants. Thus, high-pH treatment allows commercial peptide to satisfy all of the criteria for use in sensitive biophysical and cellular assays. To test whether the self-assembly mechanism of these samples was similar to that of in-house recombinant peptide, we analyzed the macroscopic self-assembly kinetics of the dataset with the greatest number of repeat experiments, previously shown in blue in Figure 4b (sample ID: 50-5-N-13), and compared our findings to those of equivalent studies in the literature.^15,75^

**Figure 9:**
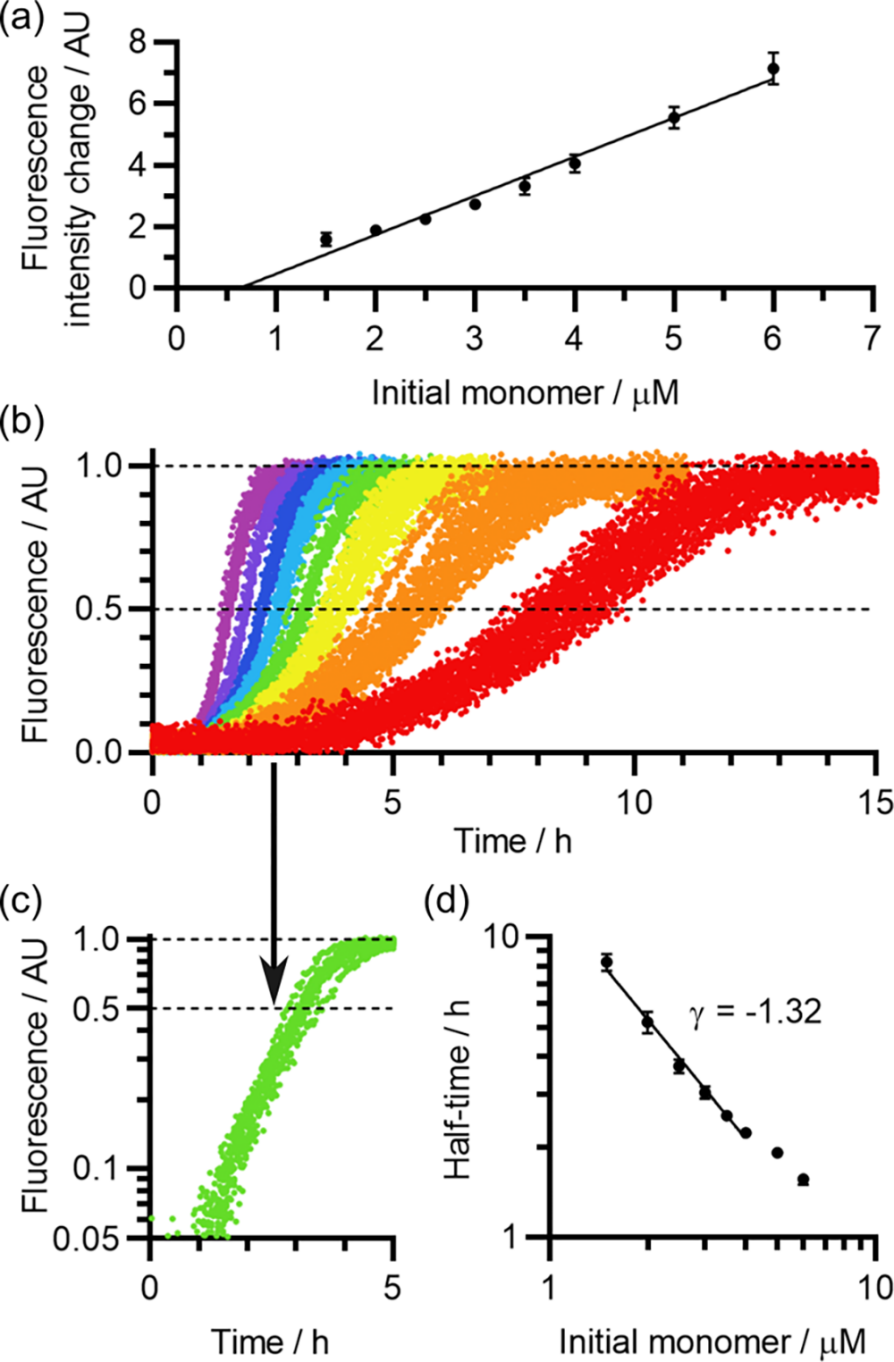
Kinetic analysis of commercial Aβ(1-42) preparations solubilized by sonication for 5 min in 50 mM NaOH, for a representative Aβ(1-42) sample. (a) Relationship between the initial monomer concentration and the normalized fluorescence intensity change across the course of ThT assays, normalized as described in Section 3.4 to allow overlaying of datasets collected with different instrument gain. Error bars represent a single standard deviation. The black line is a linear fit from 1.5 to 6.0 μM. (b) Corresponding normalized ThT fluorescence curves. The color scheme indicates the initial Aβ(1-42) concentration: red, 1.5 μM; orange, 2.0 μM; yellow, 2.5 μM; green, 3.0 μM; cyan, 3.5 μM; blue, 4.0 μM; indigo, 5.0 μM; violet, 6.0 μM. (c) The 3.0 μM data from (b), plotted on semilogarithmic axes. For clarity, only a single Aβ(1-42) concentration is shown, but the others are included in Figure S7, in which a more detailed analysis of the early-time self-assembly kinetics is provided. Note the straightening of the curve while the normalized fluorescence intensity is between 10 and 50% of its maximum, indicative of exponential kinetics in the early growth phase. (d) Concentration dependence of the mean fibrillization half-time. Error bars represent a single standard deviation. Data from 1.5 to 4.0 μM have been fitted to the equation τ_half_ = α*m*(0)^γ^, where τ_half_ is the self-assembly half-time, α is a constant of proportionality, *m*(0) is the initial Aβ(1-42) monomer concentration, and γ is the diagnostic scaling exponent as described in Section 2.8.

First, we examined the relationship between the peptide concentration and the ThT fluorescence intensity change across the course of fibrillization. As shown in Figure 9a, we observed an approximately linear relationship between the two, indicating that most of the Aβ(1-42) converted to a fibrillar state by the end of each assay, although there were small deviations from linearity that most likely reflect the impact of light scattering and lateral interactions between fibrils. As shown in Figure 9b, the curve shape itself was smooth and sigmoidal, indicating that a large number of nucleation events occurred progressively throughout the lag and growth phases. In addition, there was a well-developed lag phase, which in these contexts indicates that most fibrils are formed by secondary processes such as fragmentation or fibril-catalyzed secondary nucleation. Secondary processes amplify the number of growing fibril ends at a rate dependent on the existing fibril mass, causing exponential early-time kinetics that enhance the apparent distinction between the lag and growth phases.^87−90^ In agreement with this, as shown in Figure 9c, the early-time kinetics appear linear when plotted on semilogarithmic axes, indicating that mass accumulation is indeed exponential around that time, and secondary processes are thus occurring.

The nature and reaction order of the dominant processes responsible for generating fibrils can be inferred from the scaling exponent γ, which relates the polymerization half-time to the initial monomer concentration, τ_half_ ∝ *m*_0_^γ^. If secondary processes are negligible so that primary nucleation dominates, then γ = −*n*_c_/2, where *n*_c_ is the effective order of primary nucleation.^91,92^ If most new fibrils are formed by fragmentation of existing fibrils, then γ = −1/2.^88,89^ Finally, if most new fibrils are formed by secondary nucleation, then γ = −(*n*_2_ + 1) /2, where *n*_2_ is the effective monomer order of secondary nucleation.^90^ Under the same experimental conditions, in-house recombinant preparations typically yield γ ≈ −1.3, reflecting fibril formation via a mixture of primary and secondary nucleation, with *n*_c_ ≈ *n*_2_ ≈ 2.^15,75^ As shown in Figure 9d, applying the same procedure to our dataset gave γ = −1.32 over a range comparable to that used in Cohen et al.,^15^ almost identical to the value of γ obtained in that study. Thus, the curve shape and concentration dependence of commercial recombinant Aβ(1-42) are consistent with fibril formation due to a mixture of primary and secondary nucleation followed by elongation, with negligible fragmentation under the pseudo-quiescent conditions used in this study. This conclusion was further supported by a repeat of the same model comparison that was previously performed by Cohen et al.,^15^ which favored the same model for our dataset (Supporting Information, Section S5). Thus, properly re-solubilized commercial recombinant Aβ(1-42) has an identical self-assembly pathway to high-quality in-house preparations, and thus provides a convenient experimental alternative.

The main difference between commercial and in-house recombinant Aβ(1-42) is the overall fibrillization rate. For example, while our commercial samples have τ_half_ = 7700 ± 380 s, Silvers et al.^75^ reported τ_half_ ≈ 2000 s for in-house recombinant preparations, which is almost 4× faster. This is actually a rather small difference in the context of the Aβ(1-42) literature,^38−41^ but is still worthy of attention. Our analyses in Sections 2.3–2.6 eliminated seeding and pH-induced chemical modification as possible causes, and Silvers et al. have also shown that their peptide is highly monomeric.^39,65,75^ Therefore, the difference in rate is most likely due to differing sample composition prior to re-solubilization, particularly the abundance and variety of truncated species and post-translationally modified peptides. As previously discussed, even a small change in sequence can strongly affect Aβ(1-42) self-assembly, and small quantities of slowly aggregating variants could “poison” elongation or nucleation, while rapidly aggregating variants could template the self-assembly of other peptides in solution. Since both preparations appear to be equally monomeric and some sequence variation is inevitable in all recombinant or synthetic peptide, it is impossible to say with any confidence which source is closer in behavior to pure Aβ(1-42). However, since completely pure Aβ(1-42) does not exist *in vivo*, and both sources produce highly reproducible kinetics suitable for the same kinds of analyses, we consider this question to be of limited value anyway. Furthermore, despite the difference in rate, our kinetic analysis shows that commercial recombinant Aβ(1-42) self-assembles according to the same underlying pathway as in-house recombinant preparations. This demonstrates the generality of the underlying self-assembly pathway, and supports the use of properly re-solubilized peptide from a broad range of sources, depending on experimental convenience, to obtain high-quality mechanistic insights into Aβ self-assembly.

### 2.9 Recommendations for Solubilization of Lyophilized Aβ Samples

Solubilization is a key step in the use of Aβ from a wide range of sources, as commercial preparations are usually supplied in a lyophilized form, and in-house preparations are often lyophilized for long-term storage. Our analysis shows that high pH (>12) provides a reliable means to solubilize Aβ(1-42), yielding highly monomeric, unmodified peptide samples that exhibit extremely reproducible self-assembly kinetics upon return to near-neutral pH. This finding is relevant to users of peptide both with and without further SEC purification. For lyophilized peptide that has previously been subjected to rigorous purification procedures, high-pH treatment eliminates the need for further SEC, avoiding an inconvenient additional step that would otherwise waste around half of the sample, and result in significant dilution of the recovered peptide. For peptide that requires further rounds of SEC, either due to insufficient purity or excessive quantities of residual counterions, we expect prior high-pH treatment to significantly improve the recovery of peptide from the column compared to gentler treatments (e.g., 1 mM NaOH), by disassembling large aggregates and increasing the monomer content. In addition, we have shown that Aβ(1-42) that has been solubilized in 50 mM NaOH can be stabilized by freezing in liquid nitrogen and storage at −80 °C, preventing detectable chemical modification for a period of well over 39 days, and possibly a matter of years. This finding has broad relevance, as it means that solubilization and aliquoting can be carried out well in advance of use so that experiments can be timed more flexibly and excess peptide that is not needed for a particular experiment can be stored for future use. Our findings regarding the solubilization and storage of Aβ(1-42) are relevant to users of lyophilized peptide from all sources, as well as other isoforms such as Aβ(1-40) and disease-associated mutants. In summary, we make the following recommendations:

1. Lyophilized peptide can be effectively and reliably re-solubilized by sonication for 5 min at pH > 12. This procedure ensures high monomer yield, eliminates most pre-formed aggregates, and does not result in detectable chemical modification. Following re-solubilization, the pH should be checked as partial neutralization of the solvent may have occurred. We favor 50 mM NaOH, but similar solvents (e.g., 50 mM KOH) can be used to suit experimental constraints and retain compatibility with buffers used down the line.
2. We note that some protocols use denaturants such as urea or guanidinium hydrochloride (GuHCl) to solubilize Aβ,^12,65,93^ instead of high pH. While we have not evaluated the efficacy of these treatments, we expect a sufficiently strong denaturant to be similarly effective at monomerizing the peptide. However, the pH-based approach offers the significant advantage of allowing subsequent SEC purification to be omitted, whereas SEC is essential to exchange the buffer in cases where a denaturant has been used.
3. Peptide that has been re-solubilized to pH 12.5 (the typical end-point for 50 mM NaOH) is stable for at least 20 h at 21 °C, but should not be stored in the liquid phase for longer time periods, and, as a matter of caution, it is preferable to limit exposure to high pH as much as possible. We recommend promptly proceeding to storage at −80 °C, use in experiments, or further purification as soon as reasonably possible, although, as degradation appears to be slow, not at the expense of other experimental precautions.
4. For long-term storage, the peptide should be aliquoted, frozen by immersion in liquid nitrogen, and kept at −80 °C. This arrests pH-induced degradation and ensures long-term stability. Peptide can then be thawed for use. We note that repeatedly thawed and re-frozen samples exhibit no change in their AF4-MALS and ThT results (Supporting Information, Section S5), suggesting that thawing and re-freezing do not affect the sample. Nonetheless, we advise that such treatment be minimized as a precaution.
5. Experimenters should use a combination of techniques to assess the composition of their Aβ samples following re-solubilization, to identify any pre-formed aggregates or contaminants that may require removal. We recommend the use of a high-contrast imaging technique (e.g., NS-EM or AFM), fluorimetric assays using amyloid-sensitive dyes (e.g., ThT or Congo Red), and, crucially, a solution-phase technique capable of detecting HMW contaminants (e.g., AF4-MALS, analytical ultracentrifugation, or microfluidic separation techniques). Analytical SEC and gel-based approaches obscure HMW contaminants, and should only be used in conjunction with other techniques that are sensitive to these species.
6. If further purification is necessary, samples can be thawed and loaded onto SEC columns immediately before use. In this instance, the high-pH re-solubilization procedure still offers an advantage over gentler treatments (e.g., 1 mM NaOH), by increasing the monomer content and thus the peptide yield. If quality control analyses do not identify contaminants or aggregates that need removing, control experiments using SEC-purified samples are still advisable when switching to a new peptide source, or in instances where it is particularly important to eliminate seeding or contamination.
7. If the re-solubilized samples lack HMW aggregates, and further rounds of SEC do not affect the self-assembly kinetics, samples may be used directly without further SEC. Control experiments using SEC-purified samples should still be carried out where appropriate. In addition, it is essential that any experimental buffers should be sufficiently strong to neutralize the base added alongside the peptide, or alternatively should be pre-adjusted to reach the correct pH following this addition (Section 3.3).

### 2.10 Sources of Experimental Variation

Our results show that incomplete monomerization is a major source of experimental variation, and probably a significant contributor to the consistency issues in the literature. Since many studies use dilute base to re-solubilize Aβ,^41,46,48,53,56,57^ and few report checking the pH of the resulting samples or using analytical techniques sufficiently sensitive to detect HMW aggregates, we believe this issue may be widespread. As a result, the application of our improved solubilization procedure, or similarly aggressive treatments, is expected to significantly improve the consistency of the experimental literature. Nonetheless, as exemplified by the rate differences between the commercial recombinant Aβ(1-42) we have used and the in-house recombinant preparations that have previously been described (Section 2.8), incomplete monomerization and seeding are clearly not the only sources of experimental variation. The remainder of the variation is probably largely due to unavoidable contamination of all preparations with differing ensembles of truncated and chemically modified variants, resulting from differing preparation procedures and potentially also inter-batch variation. In addition, racemization can be an issue with peptide from synthetic sources.^94^ Even at low abundance, truncated, chemically modified, or racemized variants could strongly affect the rates of multimolecular processes they are engaged in, particularly highly structure-specific processes such as fibril elongation. Many of these modified variants are exceedingly difficult to remove; for example, deamidation of amide sidechains results in only a slight change in molecular weight (+1 Da) and racemization results in no change. Different peptide sources have distinct advantages that favor their use in different situations, meaning that variation between sources must probably be tolerated. While recombinant peptide is less prone to truncation and racemization, synthetic peptide is much more convenient, particularly for the introduction of fluorophores, cross-linking moieties, or other modifications. Similarly, while in-house recombinant preparations can be cheaper to produce when the required infrastructure is in place, commercial peptide requires less time to prepare and is more advantageous for smaller labs that have fewer resources or personnel. As a result, the use of peptide from varying sources is probably unavoidable, and the resulting variation is probably a “necessary evil” that must be borne in mind when comparing the results of different studies. Different batches prepared according to the same protocol can also exhibit variability,^95^ and this may be due to a combination of differing ease of re-solubilization and differing sequence variants between repeated preparations. While we expect our protocol to address the former, we do not expect it to address the latter. Therefore, while our protocol is able to address intra-batch variation, it is only expected to partially address inter-batch variation, although, encouragingly, we do not see clear evidence of batch dependence for our particular peptide source (Figure 2b and Table S1) (Figure 4b and Table S2). Nonetheless, despite the difference in rate between peptide sources, and the possibility that inter-batch variation may persist for some users, we have shown that proper solubilization results in a substantial improvement in internal consistency. It is also important to note that, despite comparatively small differences in rate, the underlying self-assembly pathway appears to be identical between commercial and in-house recombinant sources. Therefore, even if there are minor quantitative differences, we fully expect the qualitative and mechanistic findings of studies to be replicable between different experimental systems.

### 2.11 Concluding Remarks

In summary, we have identified incomplete solubilization as a major source of experimental variation and shown that addressing this issue through the use of more effective solvent treatments allows commercial Aβ(1-42) to be prepared to a similar standard to well-established in-house recombinant sources. In addition, our work suggests a number of other protocol modifications that will be highly convenient to users of Aβ from all sources. As most of our findings depend on the global physicochemical properties of Aβ, rather than sequence-specific effects, we fully expect these advantages to extend to other Aβ isoforms and mutants, and perhaps similar polypeptides such as islet amyloid polypeptide (IAPP) and α-synuclein. Thus, our findings will make it easier and more affordable for experimentalists to obtain high-quality samples of Aβ and other amyloidogenic polypeptides, allowing more labs to acquire large-scale, high-quality datasets describing amyloid self-assembly and activity.

## 3 Methods

### 3.1 Materials

Ultrapure recombinant Aβ(1-42) was purchased from rPeptide (Watkinsville, GA) in glass vials containing 0.5 mg (catalog number A-1163-1) or 1.0 mg (catalog number A-1163-2) lyophilized peptide, from HFIP. All other materials were analytical grade and were purchased from Fisher Scientific (U.K.) or Sigma-Aldrich (U.K.). Buffers and solvents were prepared with ultrapure deionized water (dH_2_O) and passed through a 0.2 μm filter, except where otherwise stated, and were checked frequently for the presence of dust, microbial growth, or other contaminants. Buffers and solvents for SEC and AF4-MALS were passed through a 0.1 μm filter, degassed, and prepared no more than 3 days before use.

### 3.2 Aβ(1-42) Preparation and Handling

In all preparative and experimental work, it was essential to avoid exposing Aβ(1-42) to contaminants such as dust, bubbles, and chemical residues that might affect the aggregation process. Pipetting of Aβ(1-42) solutions was performed gently, to avoid introducing bubbles that might affect aggregation. Aβ(1-42) solutions were mixed thoroughly before extraction from tubes or microplates, as larger aggregates have a tendency to sediment. Wherever possible, Aβ(1-42) was handled in low-binding Eppendorf tubes (Hamburg, Germany), to reduce the adsorption of the peptide to the interior of the tubes. Wherever possible, labware was cleaned thoroughly before use, to reduce the risk of introducing dust and other contaminants.

Prior to method optimization, lyophilized Aβ(1-42) was re-solubilized using two related protocols. In the first, Aβ(1-42) was dissolved in HFIP for aliquoting, re-lyophilized, and re-solubilized in 10 mM NaOH before use. HFIP was injected into the vials in which the peptide had been supplied using a Hamilton syringe (Reno, NV), which was kept clean by frequent washing. After the addition of solvent, vials were manually rotated for 10 s to ensure that any material on the sides came into contact with the solvent and then sonicated for 30 min using a DECON Ultrasonics sonicator bath (Sussex, U.K.). Peptide was extracted from the vials using a Hamilton syringe and split into 100 μL aliquots. The HFIP was evaporated off under a stream of N_2_ gas and the peptide was re-lyophilized and stored at −20 °C. Prior to the start of experiments, each aliquot was solubilized in 10 mM NaOH to a peptide concentration of 1 mg/mL, with trituration to ensure adequate mixing. In the second protocol, the HFIP treatment step was omitted. Each peptide sample was directly dissolved in 10 mM NaOH to a concentration of 1 mg/mL peptide, again injected into the vial using a Hamilton syringe. The vial was manually rotated for 10 s after the addition of solvent and sonicated for 30 min using a DECON Ultrasonics sonicator bath. Peptide was extracted using a Hamilton syringe, split into 50 μL aliquots in Eppendorf tubes, and rapidly frozen by immersion in liquid N_2_. Prior to use, each aliquot was thawed at 37 °C; thawing on ice was not attempted as it would prolong the time spent in the liquid phase at alkaline pH. Samples were then triturated to ensure that they were well mixed.

In the optimized protocol developed in this paper, HFIP treatment was omitted, and sonication for 30 min in 10 mM NaOH was replaced by sonication for 5 min in 50 mM NaOH, followed by rapid freezing. First, the 50 mM NaOH was injected into vials using a Hamilton syringe (Reno, NV), to a peptide concentration of 1 mg/mL. The vial was manually rotated for 10 s and sonicated for 5 min using a DECON Ultrasonics sonicator bath (Sussex, U.K.). As part of the optimization, preparations involving 0 min (i.e., without) or 30 min sonication were also attempted, but, based on the comparison in Section 2.6, and the need to balance the elimination of seeds against the risk of chemical modification, 5 min was ultimately favored. Following re-solubilization, peptide was extracted from the vials using a Hamilton syringe, split into 50 μL aliquots in Eppendorf tubes, and rapidly frozen by immersion in liquid N_2_. Prior to use, each aliquot was thawed at 37 °C; thawing on ice was not attempted as it would prolong the time spent in the liquid phase at high pH. Samples were then triturated to ensure that they were well mixed. As described in the Supporting Information (Section S5), it was found that aliquots could be repeatedly thawed and re-frozen between use, without affecting the quality of the peptide. Nonetheless, to reduce the number of experimental variables while carrying out optimization, the use of re-frozen peptide was avoided.

### 3.3 Pre-Adjusted Fibrillization Buffer

Self-assembly experiments were carried out in a 20 mM sodium phosphate buffer (pH 8) containing 200 μM EDTA, 1 mM NaN_3_, and 20 μM ThT. Sodium phosphate buffer at the same pH and ionic strength has been used in many other studies of Aβ(1-42) self-assembly (e.g., refs (15, 39, 75)), and the low ionic strength helps to reduce saturation of the various microscopic self-assembly processes,^96^ resulting in highly concentration-dependent self-assembly kinetics that is well suited for mechanistic analysis. Nonetheless, due to the comparative weakness of this buffer and the use of 10 mM NaOH or 50 mM NaOH to solubilize Aβ(1-42), it was necessary to adjust the pH of the buffer to correct for changes in pH following addition of the peptide. Correction can be carried out before or after this addition. However, if correction is carried out after the peptide has been added to the buffer stock, the peptide will already have begun to aggregate so that the delay could influence the results. In addition, adding acid to lower the pH would modify the buffer composition and increase the ionic strength from its intended value. As a better alternative, one can correct the pH in advance, by preparing a buffer stock that is more acidic than the final intended solution but reliably reaches the correct pH following the addition of a known quantity of NaOH and Aβ(1-42). Note that this correction is only necessary when preparing Aβ(1-42) solutions by direct addition of the peptide from NaOH stock and is not necessary when exchanging the peptide into the correct buffer by SEC.

#### 3.3.1 Method

Pre-adjusted fibrillization buffer containing a variable quantity of Aβ(1-42) was prepared by mixing three stocks and water, in the following ratios: 5× buffer stock containing 100 μM sodium phosphate, 5 mM NaN_3_, and 1 mM EDTA, at 20% of the final volume; 1 mg/mL (222 μM) Aβ(1-42) dissolved in 10 mM NaOH or 50 mM NaOH, at *x*% of final volume; 10 mM NaOH or 50 mM NaOH without Aβ(1-42), at (10 – *x*) % of final volume; 2 mM ThT stock, at 1% of final volume; and dH_2_O, at 69% of final volume. By varying the volume of Aβ(1-42) in 10 mM NaOH or 50 mM NaOH that was added, and always adding a complementary volume of NaOH without Aβ(1-42), it was possible to vary the final Aβ(1-42) concentration while adding a constant amount of NaOH. Since low μM concentrations of Aβ(1-42) have a negligible impact on the final pH, and the quantity of NaOH was constant, this meant the same 5× buffer stock could be used for a range of Aβ(1-42) concentrations. The ratio of NaH_2_PO_4_ and Na_2_HPO_4_ in the 5× buffer stock was chosen to give the correct pH when mixed with the NaOH and other buffer constituents, and was initially based on calculation and subsequently optimized experimentally. The final intended quantities of H_2_PO_4_^–^ and HPO_4_^2–^ can be calculated from the Henderson-Hasselbalch eq.^97^ Given an acid dissociation constant *K*_2_ ≈ 6.94 at the ionic strength of the self-assembly buffer,^98^ one expects the final self-assembly buffer to contain approximately 1.6 mM H_2_PO_4_^–^ and 18.4 mM HPO_4_^2–^. When NaOH is added to phosphate buffer, most of the hydroxide is consumed in the reaction OH^–^ + H_2_PO_4_^–^ → H_2_O + HPO_4_^2–^. Therefore, if 10 mM NaOH is incorporated into the final buffer at 10% of the final volume, 1 mM H_2_PO_4_^–^ will be converted to 1 mM HPO_4_^2–^. Thus, to achieve the correct final ratio of phosphate ions, the 5× buffer stock will need to contain 5 × (1.6 + 1.0) mM = 13 mM NaH_2_PO_4_ and 5 × (18.4 – 1.0) mM = 87 mM Na_2_HPO_4_. Similarly, if 50 mM NaOH is used, the 5× buffer stock will need to contain 5 × (1.6 + 5.0) mM = 33 mM NaH_2_PO_4_ and 5 × (18.4 – 5.0) mM = 67 mM Na_2_HPO_4_. In practice, these calculations were used to inform preparation of a test buffer stock, which was then used to prepare a test self-assembly buffer using the ratios described above. If the pH of this test solution was outside the 7.98–8.02 range, a new test buffer stock was prepared with a modified composition. The process was repeated iteratively until the test buffer stock gave a test self-assembly buffer with a pH in the 7.98–8.02 range. Once this was achieved, the modified buffer was prepared on a larger scale, tested once again, and then used in self-assembly assays. As a precaution, the pH of freshly prepared self-assembly buffers, including those containing Aβ(1-42), was checked on a regular basis. Due to the aforementioned optimization, the final pH was always in the correct range.

### 3.4 Thioflavin T (ThT) Assays

ThT assays were carried out in a 20 mM sodium phosphate buffer (pH 8) containing 200 μM EDTA, 1 mM NaN_3_, and 20 μM ThT. Prior to use, Aβ(1-42) was re-solubilized to a concentration of 1 mg/mL peptide in 10 or 50 mM NaOH, as described in Section 3.2. In most instances, this was then mixed with concentrated buffer stock, a complementary volume of NaOH, ThT stock, and dH_2_O, as described in Section 3.3, to obtain a solution of 1–10 μM Aβ(1-42) in the aforementioned fibrillization buffer. In cases where the peptide was purified by SEC following re-solubilization, purification was carried out in a 20 mM sodium phosphate (pH 8.0), 200 μM EDTA, 1 mM NaN_3_ mobile phase so that eluent was simply collected on ice, diluted in buffer and supplemented with ThT, as described in Section 3.7. Both methods ultimately yielded 1–10 μM Aβ(1-42) in 20 mM sodium phosphate buffer (pH 8) containing 200 μM EDTA, 1 mM NaN_3_, and 20 μM ThT. This was mixed thoroughly, taking care not to introduce bubbles, and pipetted into the wells of a low-binding 96-well microplate (Corning 3881, NY), with 100 μL per well. Experiments were typically performed with 5 replicate wells per Aβ(1-42) concentration, as well as 5 blank wells that contained the same solution without Aβ(1-42), although some experiments had a smaller number of replicates. In all cases, the scale of experiments was planned so that the dead time was small compared to the self-assembly timescale (<300 s). After pipetting, the plate was sealed with a qPCR seal (4titude, U.K.) to restrict evaporation, and incubated in a FLUOstar Omega plate reader (BMG Labtech, U.K.) at 37 °C. Fluorescence readings were taken every 2 min, with 4 s double-orbital shaking (100 rpm) before reading to dislodge any aggregates weakly associated with the sides or bottom of the plate wells. Based on previous analysis of the effects of shaking on Aβ(1-42) self-assembly,^15^ this amount of shaking would not be expected to induce significant fragmentation of fibrils, a conclusion that was supported by the high concentration dependence of the self-assembly kinetics (Figure 9), and the more detailed analysis of those kinetics in the Supporting Information (Section S5). ThT fluorescence was measured with an excitation wavelength of 440 nm and an emission wavelength of 485 nm. For data presentation, raw fluorescence intensities were baselined by subtracting the average fluorescence intensity of the blank wells from the same experiment. Where normalization was carried out, this was achieved by dividing the baseline-subtracted fluorescence intensities by the maximum average fluorescence intensity of all comparable replicate wells.

### 3.5 Asymmetric Flow Field-Flow Fractionation (AF4)

Samples were analyzed using an AF4 frit inlet (FI) channel equilibrated in 1 mM NaOH, with a 250 μm spacer and a 1 kDa PES membrane, and in-line UV (280 nm; Shimadzu, U.K.), MALS, and RI detectors. Except where otherwise stated, the channel, associated pumps, autosampler, and detectors were purchased or loaned from Postnova Analytics (Landsberg am Lech, Germany). The RI detector was purged following equilibration, and the UV and RI detectors were then zeroed. The sample, which consisted of 1 mg/mL Aβ(1-42) re-solubilized in either 10 mM NaOH or 50 mM NaOH, was loaded into the autosampler and 20 μL was injected into the channel. The sample was separated in a 1 mM NaOH mobile phase, with 0.2 mL/min TIP and detector flow; the focus and cross-flow rates were also matched to one another and varied throughout the run. To ensure that results were comparable, after initial optimization, the same flow profile was used for all runs: cross-flow began at 4.5 mL/min for 20 min, followed by a linear decay to 0 mL/min over the course of 10 min, followed by a period with constant cross-flow of 0 mL/min for at least 30 min. Blanks consisting of the same solvent without Aβ(1-42) were run before and after samples, to check that the channel was clean and to allow blank subtraction of the UV and RI detector signals.

Data processing and estimation of sample concentration, molecular weight, and recovery were carried out in the AF2000 Control software (Postnova Analytics, Landsberg am Lech, Germany), with additional data processing in GraphPad Prism version 8.3.0. For Aβ(1-42) in a 1 mM NaOH mobile phase, we used a UV_280_ extinction coefficient of 1860 M^–1^ cm^–1^, which was determined empirically by UV absorbance spectrometry of filtered tyrosine at a range of pH values, confirmed by later spectroscopy measurements, and accounts for ∼76% deprotonation of Tyr10 at pH 11 (Figure S11a). We note that, although there is an isosbestic point nearby at 276 nm, the relatively rapid decrease in tyrosine absorbance and increase in tyrosinate absorbance to the right of this point mean that deprotonation still has a significant effect on the extinction coefficient at 280 nm. As a result, our tyrosine and tyrosinate spectra are close to previously reported spectra for Aβ(1-16), which suggest a similar extinction coefficient and fold-change due to deprotonation.^99^ Full-length Aβ(1-42) spectra exhibit clear signs of light scattering for peptide prepared in 10 mM NaOH (final pH 10.0), but also a lesser degree of light scattering for peptide prepared in 50 mM NaOH (final pH 12.5), likely due to the HMW species that eluted after cross-flow in our AF4-MALS runs (Figure S11b). As a result, the tyrosine/tyrosinate extinction coefficient is more appropriate for analysis of the monomer and oligomer fractions in our AF4-MALS runs. Molecular weight values were calculated by fitting models to the UV_280_ and MALS signals. The molecular weights in Figure 2e were obtained using a fourth-degree Berry fit from 2.0 to 7.0 min, a linear Zimm fit from 7.0 to 12.5 min, and a third-degree Berry fit from 28.0 to 50.0 min. The molecular weights in Figure 3c were obtained using a linear Zimm fit. To obtain the approximate molecular weight distribution in Figure 3d, a cumulative density function *P*(*M*_w_ ≤ *M*) was calculated first, which gives the proportion of sample that eluted in fractions with molecular weight *M*_w_ less than or equal to *M*. This was approximated using the summation
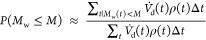
1where *t* is the retention time of each reading, Δ*t* is the interval between readings, *M*_w_(*t*) is the instantaneous molecular weight of the eluent at each retention time, *V̇*_d_(*t*) is the volumetric flow rate through the detector, and ρ(*t*) is the UV_280_-derived concentration of Aβ(1-42) in the eluent, where *M*_w_(*t*), *V̇*_d_(*t*), and ρ(*t*) are all functions of time. This summation tends toward an exact integral as Δ*t* → 0, and the experimental parameter of Δ*t* = 0.12 min was sufficiently small for the approximation to be reasonably accurate. The probability density function *p*(*M*), which gives the probability density of species with mass *M*, can be calculated as *p*(*M*) = d*P*(*M*_w_ ≤ *M*)/d*M*. However, when presenting the data on a logarithmic axis, peaks in the probability density function are horizontally stretched at low *M* and horizontally compressed at high *M*, meaning that the peak area is no longer proportional to the mass of material. Therefore, in Figure 3d, we used the logarithmically corrected probability density function *Mp*(*M*) ≡ d*P*(*M*_w_ ≤ *M*)/*d* log *M*, which ensures that the peak area in the figure is exactly proportional to the relative quantity of material in the peaks.

### 3.6 Negative-Stain Electron Microscopy (NS-EM)

Prior to negative staining, Aβ(1-42) was diluted to a concentration of 4 μM peptide in a pre-adjusted fibrillization buffer containing 20 mM sodium phosphate (pH 8.0), 200 μM EDTA, 1 mM NaN_3_, and 20 μM ThT, according to the same protocol that was used to prepare Aβ(1-42) for ThT assays (Section 3.3). The resulting sample was deposited on grids immediately, or after incubation for 1 h in a 96-well plate treated with a PEG-like low-binding surface (Corning 3881, NY), at 37 °C. Carbon-coated grids were glow-discharged at low pressure in the glow discharge unit of a Cressington 208 carbon coater (Ted Pella, Inc., CA). Samples were mixed gently and 7 μL was pipetted onto a glow-discharged grid and left to adsorb for 1 min. Grids were blotted edge-on with filter paper, and briefly washed twice in dH_2_O and once in 0.75% uranyl formate stain, blotting after each wash. Grids were then immersed in 0.75% uranyl formate stain for 20 s, blotted again, and dried with a vacuum pump. Grids were imaged on a Philips CM100 TEM at 100 kV, with either a LaB_6_ cathode or tungsten filament. Micrographs were recorded with a 1024 × 1024 px Gatan CCD camera, and images were analyzed using FIJI.^100,101^

### 3.7 Size Exclusion Chromatography (SEC)

Samples were analyzed and purified in small batches using an analytical Superdex 75 column (GE Healthcare) equilibrated in 20 mM sodium phosphate buffer (pH 8) with 200 μM EDTA and 1 mM NaN_3_. The column was run with the TIP pump from the AF4 system (Postnova Analytics, Landsberg am Lech, Germany), and in-line UV (280 nm; Shimadzu, U.K.), MALS (Postnova Analytics, Landsberg am Lech, Germany), and RI (Postnova Analytics, Landsberg am Lech, Germany) detectors. The RI detector was purged following equilibration, and the UV and RI detectors were then zeroed. The sample was injected, with an injection volume of 50 μL, and run at 1.0 mL/min for 35 min. Between sample runs, blanks consisting of the same solvent without the Aβ(1-42) were loaded to check that the column was clean and to allow blank subtraction of the detector signals. Data processing and estimation of sample concentration, molecular weight, and recovery were carried out in the AF2000 Control software (Postnova Analytics, Landsberg am Lech, Germany), with final data processing in GraphPad Prism version 8.3.0. For preparative runs, the sample was collected at the end. Purified Aβ(1-42) was collected on ice between 15.3 and 16.3 min after injection, corresponding to an elution time of 13.6–14.6 min. Eluted Aβ was mixed and split into three aliquots; these were then diluted to 60, 80, or 100% (i.e., undiluted) their concentration in the same elution buffer and supplemented with 20 μM ThT from a 2 mM stock, as described in Hellstrand et al.^39^ This yielded solutions containing approximately 3.8, 5.0, or 6.3 μM Aβ(1-42) as determined by RI, in almost exactly 20 mM sodium phosphate (pH 8), 200 μM EDTA, and 1 mM NaN_3_ (99.0% nominal concentration), with exactly 20 μM ThT. The pH of these samples was confirmed experimentally. Due to the potential for ThT to interact with the column, ThT had to be added from a concentrated stock after purification; the slight dilution of the buffer due to the addition of 1% ThT is too small to significantly affect the kinetics, and cannot explain any significant differences between these experiments and corresponding experiments performed with exactly 20 mM sodium phosphate, 200 μm EDTA, and 1 mM NaN_3_. Purified Aβ(1-42) was used immediately in ThT assays, and exact Aβ(1-42) concentrations accounting for all dilutions were calculated retrospectively from the RI quantitation data, after the start of the ThT experiment.^39^

## Supporting Information Available

Section S1: Summary of Aβ(1-42) samples re-solubilized in 10 mM NaOH (Table S1) and 50 mM NaOH (Table S2), including a key to the color coding of these samples in Figures 2–4 and 8; Section S2: Additional analysis of AF4-MALS and ThT data, including the cumulative molecular weight distribution of high-pH Aβ samples (Figure S1), further analysis of the kinetic consistency of high-pH Aβ samples (Figure S2), and an analysis of the effect of sonication time on the self-assembly kinetics (Figure S3); Section S3: AF4-MALS and ThT analysis of the effects of removing HMW material by ultracentrifugation (Figure S4); Section S4: Analysis of 50 mM NaOH Aβ preparations by LC–MS (Figures S5 and S6); Section S5: Further kinetic analysis of Aβ(1-42) self-assembly mechanisms, including fitting of exponential-like early-time kinetics (Figure S7), global fitting comparison to determine the self-assembly mechanism (Figure S8, Table S3), and individual fitting of self-assembly curves (Figure S9); Section S6: Re-freezing of Aβ solubilized in 50 mM NaOH (Figure S10); Section S7: Absorbance spectroscopy of tyrosine and Aβ(1-42) (Figure S11) (PDF)
