# Supplementary material for: Simple, Reliable Protocol for High-Yield Solubilization of Seedless Amyloid-β Monomer
Source: ACS Chem Neurosci. 2022 Dec 13;14(1):53–71. doi: 10.1021/acschemneuro.2c00411 (PMC9817077; doi:10.1021/acschemneuro.2c00411)
Supplement: Supplementary file 1 — cn2c00411_si_001.pdf [file cn2c00411_si_001.pdf]

A Simple, Reliable Protocol For High-Yield Solubilization of Seedless Amyloid-β Monomer (Supporting Information)

[1] Alexander I. P. Taylor, Peter J. Davis, Liam D. Aubrey, Joshua B. R. White, Zoe N. Parton, and Rosemary A. Staniforth*

School of Biosciences, University of Sheffield, Sheffield, S10 2TN, United Kingdom

E-mail: r.a.staniforth@sheffield.ac.uk

S1 Key to resolubilized Aβ(1-42) samples

Table S1: Summary of Aβ(1-42) samples resolubilized in 10 mM NaOH. Each sample ID corresponds to a resolubilization carried out on a different date. The color schemes used for the overlays in Fig. 2 are indicated in the right-hand columns; where more than one color is listed, each color is a different repeat experiment with the same sample.

**Sample ID**	**Batch**	**[NaOH]**	**Sonication**	**HFIP step**	**Color code**	
					Fig. 2(b)	Fig. 2(c-f)
10-30-H-1	1	10 mM	30 min	yes	red	—
10-30-H-2	1	10 mM	30 min	yes	orange	—
10-30-H-3	1	10 mM	30 min	yes	yellow	—
10-30-H-4	1	10 mM	30 min	yes	green	—
10-30-N-1	2	10 mM	30 min	no	cyan	—
10-30-N-2	2	10 mM	30 min	no	blue	—
10-30-N-3	2	10 mM	30 min	no	indigo	—
10-30-N-4	2	10 mM	30 min	no	violet	—
10-30-N-5	3	10 mM	30 min	no	gray	red
10-30-N-6	3	10 mM	30 min	no	—	amber
10-30-N-7	2	10 mM	30 min	no	—	green, cyan, blue
10-30-N-8	3	10 mM	30 min	no	—	indigo

Table S2: Summary of Aβ(1-42) samples resolubilized in 50 mM NaOH. Each sample ID corresponds to a resolubilization carried out on a different date. The color schemes used for the overlays in Fig. 3, 4, and 8 are indicated in the right-hand columns; where more than one color is listed, each color is a different repeat experiment with the same sample.

**Sample ID**	**Batch**	**[NaOH]**	**Sonication**	**HFIP step**	**Color code**		
					Fig. 3a-c	Fig. 4b	Fig. 4c, Fig. 8
50-5-N-1	3	50 mM	5 min	no	red	—	—
50-5-N-2	3	50 mM	5 min	no	orange	—	—
50-5-N-3	3	50 mM	5 min	no	yellow, green, indigo	—	orange
50-5-N-4	3	50 mM	5 min	no	cyan, blue	—	yellow
50-5-N-5	3	50 mM	5 min	no	violet	—	green
50-5-N-6	3	50 mM	5 min	no	gray	—	—
50-5-N-7	3	50 mM	5 min	no	black	—	—
50-5-N-8	3	50 mM	5 min	no	red	—	—
50-5-N-9	3	50 mM	5 min	no	orange	—	—
50-5-N-10	2	50 mM	5 min	no	—	red	—
50-5-N-11	3	50 mM	5 min	no	—	amber	cyan
50-5-N-12	3	50 mM	5 min	no	—	green	blue
50-5-N-13	3	50 mM	5 min	no	—	blue	indigo
50-5-N-14	3	50 mM	5 min	no	—	—	red
50-5-N-15	3	50 mM	5 min	no	—	—	violet
50-0-N-1	3	50 mM	—	no	—	—	—
50-0-N-2	3	50 mM	—	no	—	—	—
50-0-N-3	3	50 mM	—	no	—	—	—
50-30-N-1	3	50 mM	30 min	no	—	—	—
50-30-N-2	3	50 mM	30 min	no	—	—	—
50-30-N-3	3	50 mM	30 min	no	—	—	—

S2 Additional analysis of AF4-MALS and ThT data

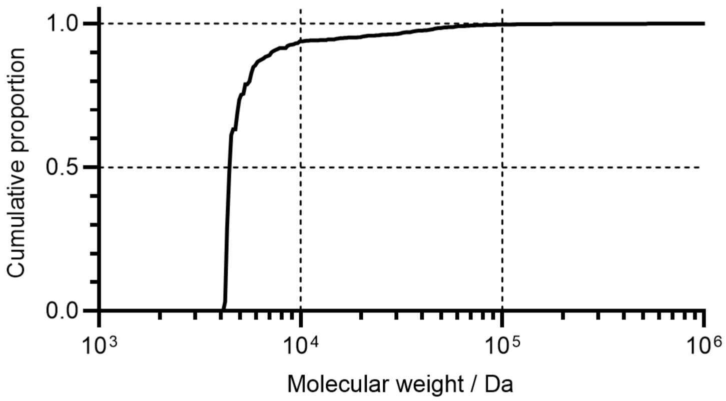

Figure S1: The approximate cumulative molecular weight distribution of Aβ(1-42) samples prepared by sonication for 5 min in 50 mM NaOH, obtained by AF4-MALS. This distribution is derived from analysis of the ROI in Figure 3(c), and was used to obtain the logarithmically corrected molecular weight distribution shown in Figure 3(d), in the main text. The black line shows the cumulative proportion of sample that eluted in AF4 fractions with an average molecular weight M_w less than or equal to the value indicated on the x-axis.

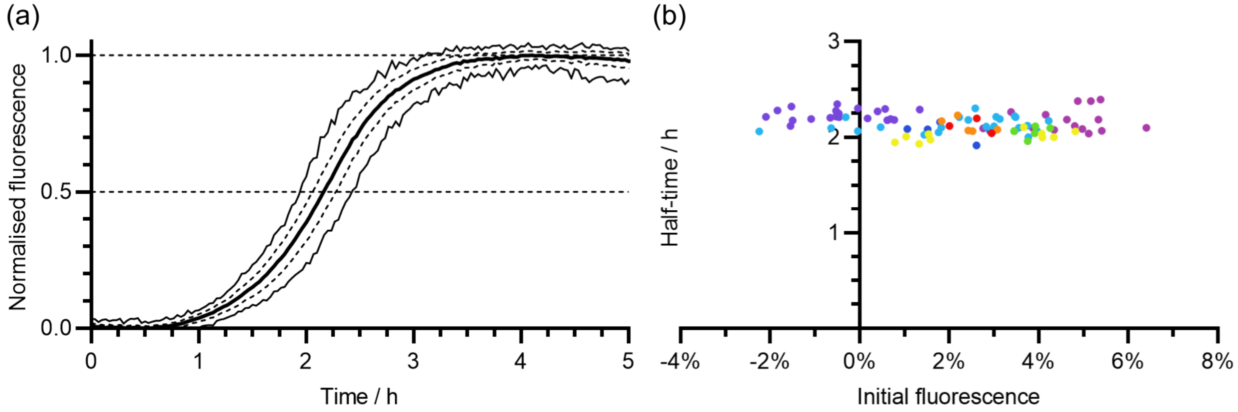

Figure S2: Kinetic consistency of 4 µM Aβ(1-42) solubilized by sonication for 5 min in 50 mM NaOH prior to use in ThT assays. Self-assembly was induced by dilution of the high-pH Aβ(1-42) sample into a pH-corrected 20 mM sodium phosphate buffer (pH 8) containing 200 µM EDTA, 1 mM NaN_3, and 20 µM ThT, at 37°C. (a) Consistency of the self-assembly curves. The black central curve is the mean, while the dashed curves represent the mean plus or minus a single standard deviation, and the thin black curves at the edges show the range. (b) The initial ThT fluorescence does not correlate with the fibrillization half-time, showing that seeding does not explain variation in the latter. The summary statistics in both panels are derived from the data presented in Figure 4(c) in the main text. Panel (b) uses the same color scheme as Figure 4(c), with each color indicating a different peptide sample re-solubilized on a separate occasion.

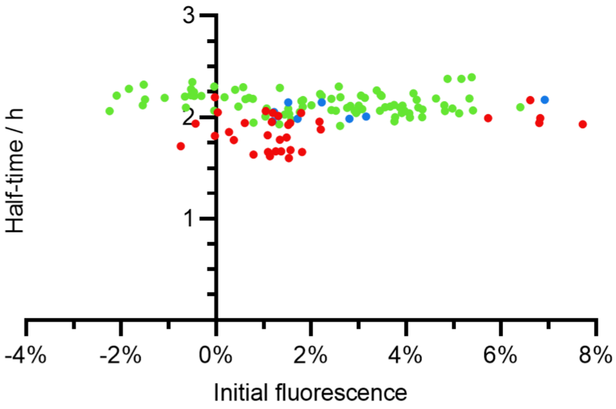

Figure S3: Effect of sonication time on the self-assembly half-time and initial ThT fluorescence of Aβ(1-42) preparations re-solubilized by sonication in 50 mM NaOH prior to use in ThT assays. Self-assembly was induced by dilution of the high-pH Aβ(1-42) sample into a pH-corrected 20 mM sodium phosphate buffer (pH 8) containing 200 µM EDTA, 1 mM NaN_3, and 20 µM ThT, at 37°C. The initial fluorescence is given as a percentage of the final fluorescence at the end of self-assembly. The color scheme is the same as that used in Figure 7(a-c): red, no sonication; red, 0 min (no sonication); green, 5 min; blue, 30 min. Derived from the self-assembly curves presented in Figure 7(a-c) in the main text.

S3 Removal of HMW material from 50 mM NaOH Aβ preparations by ultracentrifugation

As described in the main text, our NS-EM and SEC experiments did not identify fibril seed in Aβ(1-42) samples re-solubilized by sonication for 5 min in 50 mM NaOH, and showed that attempts to remove any putative seed or oligomers had no impact on the self-assembly kinetics. To complement this, and further explore the nature of the HMW contaminant that eluted after cross-flow in AF4 runs, we removed the HMW material by ultracentrifugation and examined the effect on the self-assembly kinetics. Firstly, an Aβ(1-42) sample was re-solubilized to a concentration of 1 mg/ml peptide by sonication for 5 min in 50 mM NaOH. An aliquot of this sample was then diluted 10× in dH_2O to give 0.1 mg/ml Aβ(1-42) in 5 mM NaOH, centrifuged at 436,000*g* for 1 h, and examined by AF4-MALS and ThT assays. The dilution was carried out to obtain a sufficiently large volume for ultracentrifugation. As a control, another aliquot from the same re-solubilized sample was diluted in the same manner, and incubated in a centrifuge tube for the same period of time without ultracentrifugation. Both samples were kept at 21°C during ultracentrifugation/incubation. For the AF4-MALS experiments, a larger-than-usual injection volume of 200 µl was used to ensure the same mass injection as other experiments with undiluted Aβ(1-42), which caused a small peak shift in the elugram. For the ThT assays, the buffer was pre-adjusted to account for the increased volume of Aβ(1-42) stock, and the pH of the dissolved solution was checked.

As shown in Figure S4, ultracentrifugation resulted in an approximately 3× reduction in the integral of the UV signal after cross-flow; in the control, the integrated HMW signal was equivalent to 1.004 µg Aβ(1-42) (although it is not certain that this material was Aβ(1-42), so the true mass is unknown), whereas in the centrifuged sample the integral was equivalent to 0.365 µg Aβ(1-42). Similarly, ultracentrifugation caused an approximately 4× reduction in the integral of the LS 90° signal. Although the composition and degree of homogeneity of the HMW material is not known, making exact quantitation difficult, the AF4 data indicate

that the majority of this material was removed. Despite removal of this material, the treated and untreated samples exhibited almost identical ThT kinetics, indicating that the HMW material did not cause a significant level of seeding. One possibility is that the HMW fraction consists of off-pathway Aβ(1-42) species. However, the fact that the full expected 20 µg of Aβ(1-42) was recovered in the LMW fraction favours the alternative possibility, that the HMW material is not actually Aβ(1-42). Instead, it may consist of small quantities of dust or pre-existing contaminants in the vials, which were resistant to 50 mM NaOH but had no observable seeding potential.

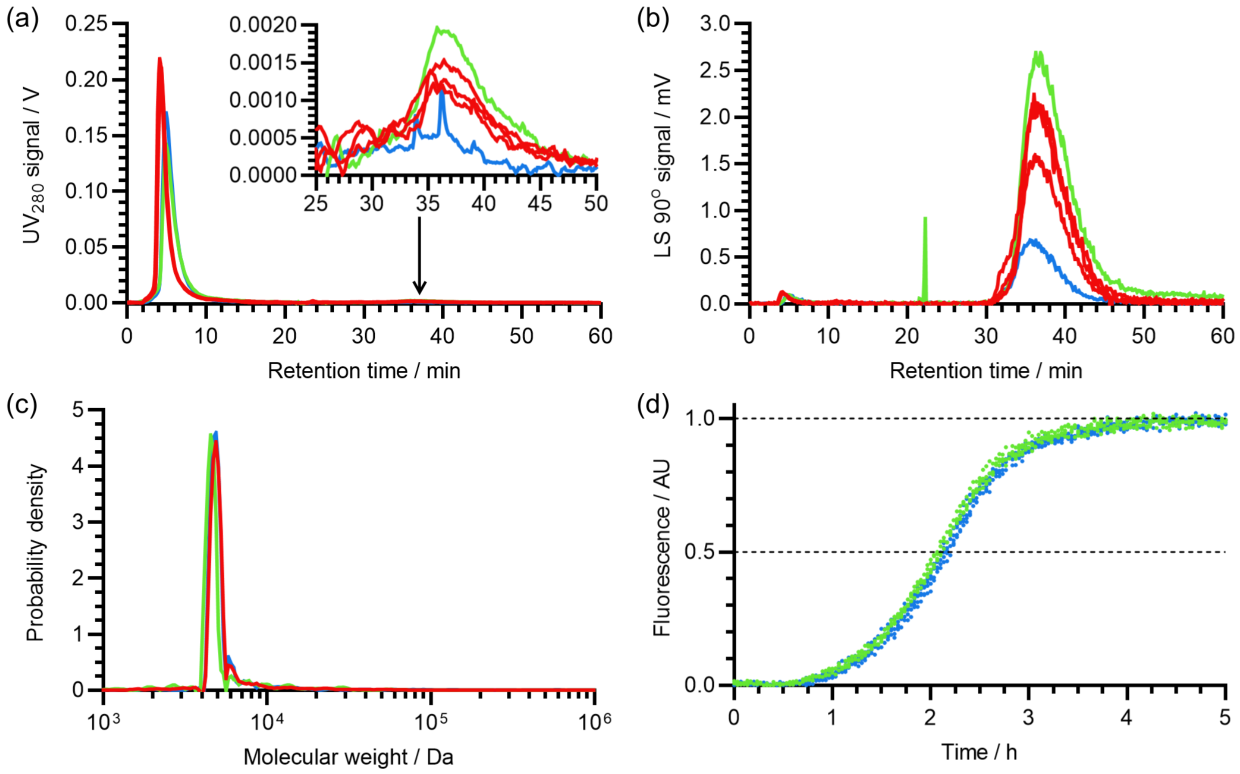

Figure S4: Removal of HMW material from high-pH Aβ(1-42) preparations does not affect the self-assembly kinetics. Panels show (a) the UV_{280} signal, (b) the LS 90° signal, (c) the approximate M_w distribution, derived from MALS analysis of the ROI from 3.3-12.0 min using a linear Zimm fitting approach, and (d) the corresponding ThT self-assembly kinetics of an Aβ(1-42) sample prepared in 50 mM NaOH. Color scheme: red, 1 mg/ml sample before dilution (not included in ThT assay); green, 10× diluted 0.1 mg/ml sample, incubated for 1 h before use in experiments; blue, 10× diluted 0.1 mg/ml sample, centrifuged at 436,000g for 1 h and then used in experiments.

S4 Analysis of 50 mM NaOH Aβ preparations by LC-MS

In the main text, our AF4-MALS, ThT, NS-EM, and SEC-MALS/ThT experiments showed that Aβ(1-42) re-solubilized by sonication in 50 mM NaOH is predominantly monomeric and lacks fibril seed. However, our MALS experiments did not provide sufficient M_w resolution and accuracy to identify chemical modifications, truncated Aβ variants, and contaminants with a similar molecular weight. Therefore, Aβ samples re-solubilized by 5 min or 30 min sonication were examined by liquid chromatography mass spectrometry (LC-MS). Samples were separated in an Agilent Zorbax Extend-C18 reverse-phase column with a 1.8 µm particle size, equilibrated with 0.1% formic acid and 5% acetonitrile. The injection volume was 1 µl, and the sample was eluted at 0.4 ml/min, with a linear acetonitrile gradient from 5% to 95% over 15 min. Masses were detected using an Agilent 6530 Q-ToF (Agilent Technologies, Santa Clara, CA) in ESI positive ion mode. Figure S5 shows the reverse-phase HPLC elugram of an Aβ sample that had been sonicated for 5 min, and Figure S6 shows the equivalent data for an Aβ sample that had been sonicated for 30 min. Both samples gave very similar results, and no detectable degradation occurred during the longer sonication. The mass spectrum contained a strong peak at 4513 Da, corresponding to monomeric Aβ(1-42), as well as a number of smaller peaks in the 2500-5000 Da range, which likely corresponded to contaminants and truncated variants. The only other species that could be identified from its molecular weight alone was the secondary peak at 3895 Da, whose molecular weight was consistent with the N-terminally truncated Aβ(6-42) variant. Thus, the mass spectrum indicates that the peptide samples consist predominantly of Aβ(1-42), with a small but significant quantity of Aβ(6-42), and smaller amounts of chemically modified, truncated, or non-Aβ species. This heterogeneity appears to have been present in the vials prior to solubilisation, as there is no progressive change in sample quality during the high pH treatment, as judged by ThT assays and comparison of the 5 min and 30 min LC-MS data. The loss of the 5 N-terminal residues (DAEFR) in the Aβ(6-42) variant is unlikely to profoundly affect the self-assembly process as the central and C-terminal regions of the peptide are believed to be more important in

initiating and stabilizing fibril structures (*1–7*). Nonetheless, some studies have supported a supplementary role for the N-terminus in modulating the aggregation rate, toxicity, and mechanical properties of fibrils (*8–11*), so the presence of a small quantity of Aβ(6-42) may explain some kinetic differences between this peptide and Aβ from other sources. Overall, the degree of heterogeneity in the commercial peptide is similar to that observed for in-house recombinant preparations, which also contain contaminants and truncated variants (*12*). As with oligomerization, some level of contamination and truncation is impossible to eliminate, and does not necessarily affect the validity or reliability of experimental results.

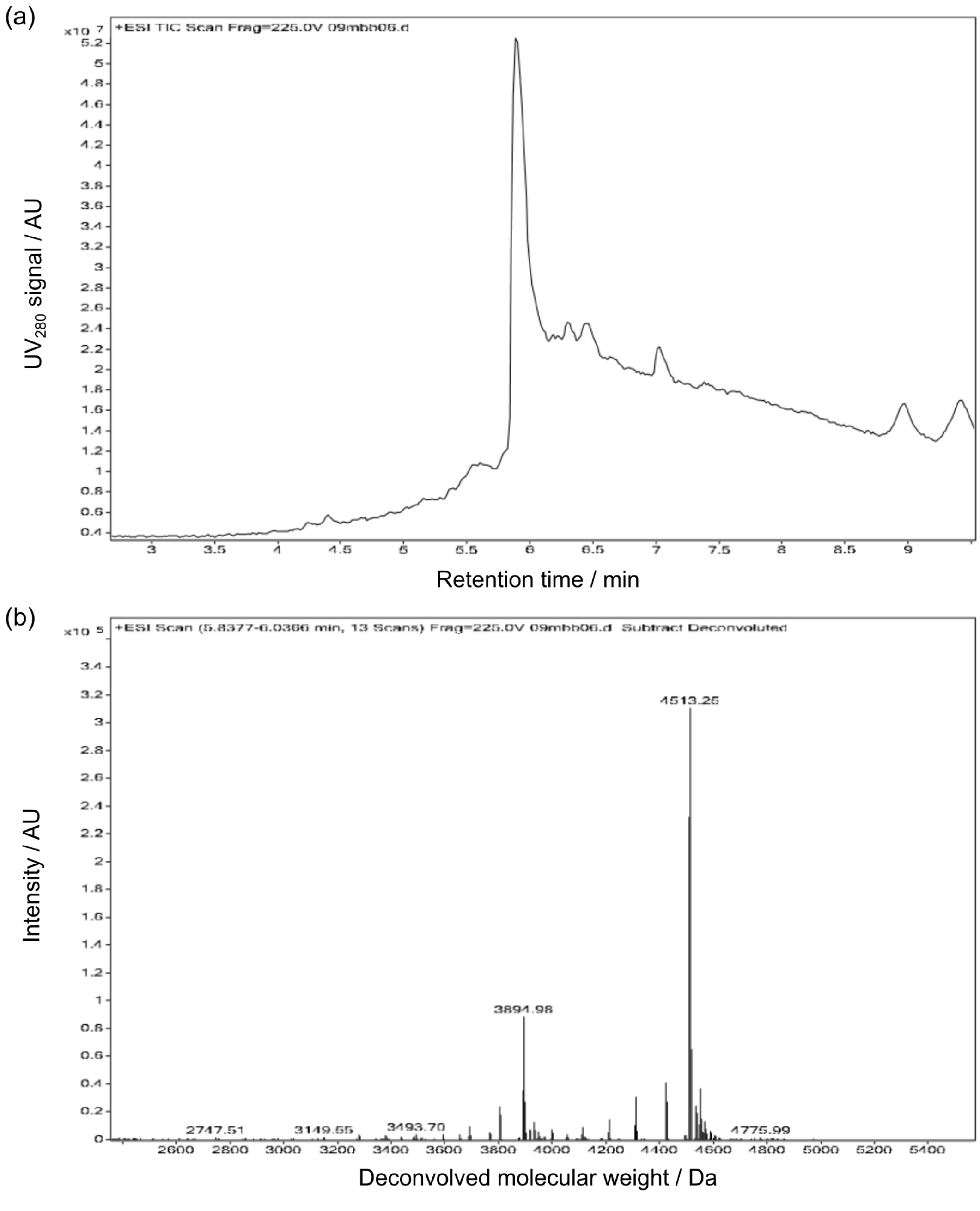

Figure S5: LC-MS of Aβ(1-42) prepared by 5 min sonication in 50 mM NaOH. (a) Reverse-phase HPLC elugram. (b) Deconvolved mass spectrum of the reverse-phase HPLC fraction collected from 5.84-6.04 min.

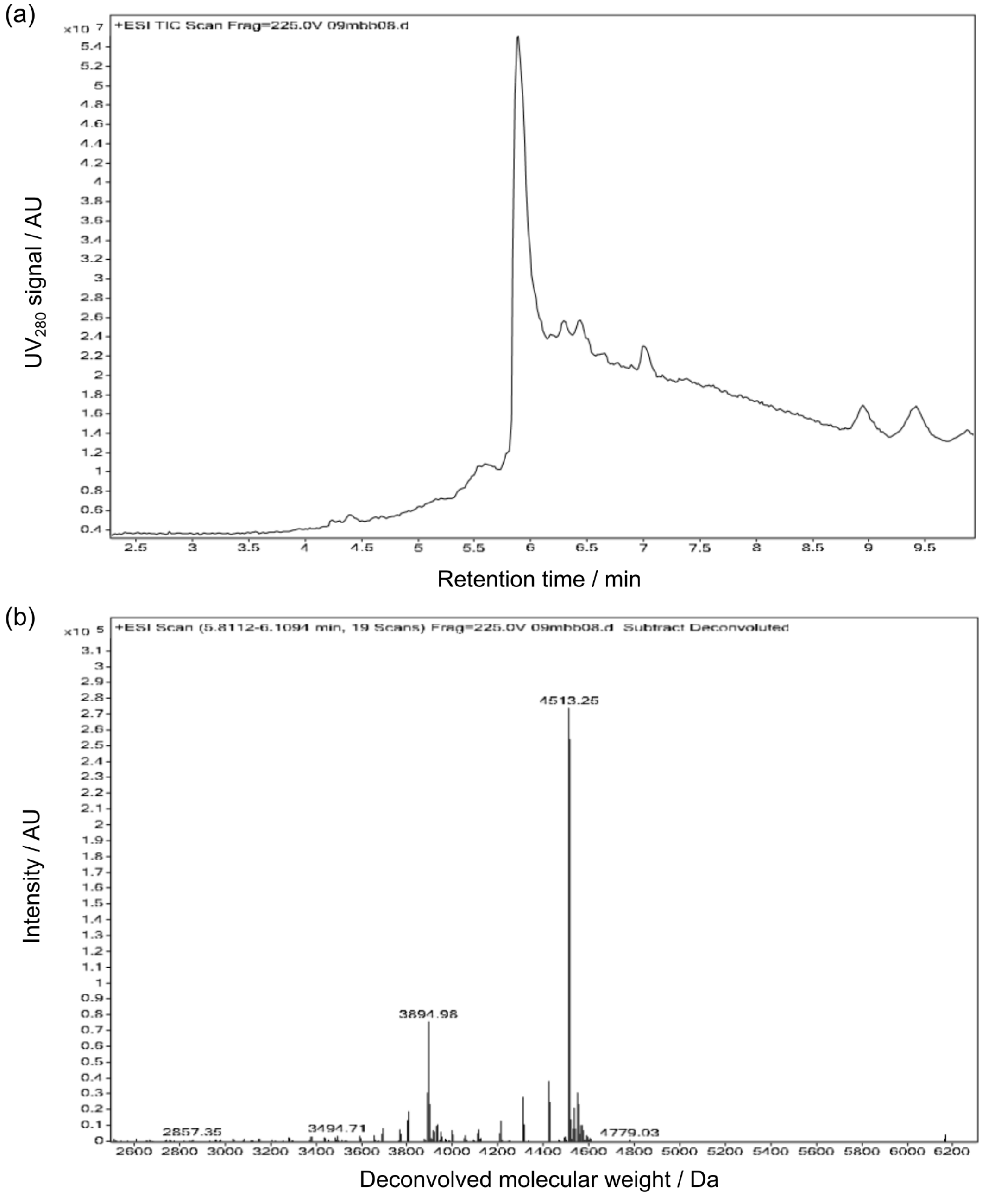

Figure S6: LC-MS of Aβ(1-42) prepared by 30 min sonication in 50 mM NaOH. (a) Reverse-phase HPLC elugram. (b) Deconvolved mass spectrum of the reverse-phase HPLC fraction collected from 5.81-6.11 min.

S5 Kinetic analysis of Aβ(1-42) self-assembly mechanisms

In the main text, we analyzed the self-assembly kinetics of Aβ(1-42) samples that had been prepared by sonication for 5 min in 50 mM NaOH, and used in ThT assays in a pH-adjusted 20 mM sodium phosphate (pH 8) buffer containing 200 µM EDTA, 1 mM NaN_3, and 20 µM ThT. We concluded that the fibrillization curves of these samples exhibited exponential early-time scaling, as evidenced by linearity when plotted on semi-logarithmic axes and a half-time concentration-dependence very close to that previously described in the literature, consistent with the established self-assembly pathway where fibrils are produced by a combination of primary and secondary nucleation, with reaction order $n_c \approx n_2 \approx 2$. In this section, we present a more detailed kinetic analysis that supports this conclusion.

Firstly, to more quantitatively test whether the fibrillization curves exhibited the expected early-time self-assembly kinetics, we individually fitted the same self-assembly curves that were analysed in Section 2.8 of the main text with the equation (*13–15*)

$$\frac{M(t)}{m(0)} = \frac{\lambda^2}{2\kappa^2}\left(e^{\kappa t} + e^{-\kappa t} - 2\right), \tag{S1}$$

which is correct for nucleated polymerization with a directly fibril-catalyzed secondary process in the early part of the self-assembly curve, before monomer depletion effects become significant. Here, $M(t)$ is the concentration of Aβ(1-42) monomers incorporated into amyloid fibrils as a function of time, $m(t)$ is the free monomer concentration, subject to the conservation law $m(0) = m(t) + M(t)$ in the absence of fibril seed, and λ and κ are macroscopic rate constants that describe the effective turnover rate of primary and secondary nucleated polymerization pathways, and whose precise, model-specific definitions will be given later in this section. The fits, shown on semi-logarithmic axes in Figure S7, confirmed that the early time self-assembly kinetics were consistent with a mechanism involving primary nucleation and a secondary fibril-generating process, with the latter becoming more important as the fibril mass increases, and resulting in a straightening of the curve when viewed on

double-logarithmic axes.

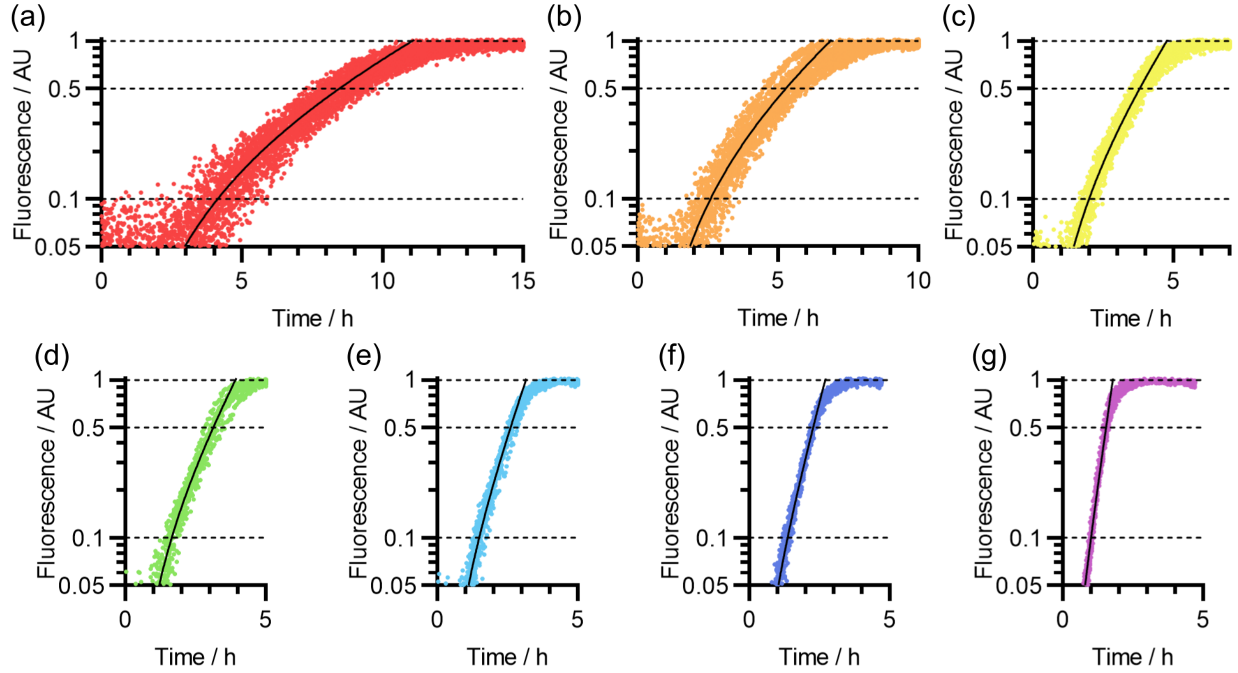

Figure S7: The early-time kinetics of Aβ(1-42) preparations solubilized by sonication for 5 min in 50 mM NaOH are consistent with a mechanism involving a mixture of primary and secondary nucleation. The data shown in panels (a-g) are the same data previously presented in Figure 9 in the main text, split according to the initial Aβ(1-42) concentration: (a) red, 1.5 µM; (b) orange, 2.0 µM; (c) yellow, 2.5 µM; (d) green, 3.0 µM; (e) cyan, 3.5 µM; (f) blue, 4.0 µM; (g) violet, 6.0 µM. In each panel, the black curve is Eq. S1, fitted to the normalized fluorescence intensities up to the half-time. Note the initial curvature, reflecting dominant primary nucleation, followed by a straightening of the curve due to increasing secondary nucleation, which results in exponential self-assembly kinetics. The model later departs from the data, as the early-time solution does not account for monomer depletion.

Statistical comparison of the ability of candidate models to fit entire time courses provides a powerful and complementary approach to deduce the self-assembly mechanism. In Cohen *et al.* (*16*), nonlinear regression of Aβ(1-42) self-assembly curves supported a nucleated polymerization model with autocatalytic secondary nucleation, discounting alternative scenarios where there was no secondary process, or the secondary process was fragmentation. Since then, data obtained using Aβ(1-40) and higher concentrations of Aβ(1-42) have suggested a modified, multi-step secondary nucleation mechanism which is saturable and exhibits Michaelis-Menten-like kinetics; this modification extends the model's range to include

higher Aβ(1-42) concentrations (*17*, *18*). To test whether an equivalent analysis favored saturable secondary nucleation for our Aβ(1-42) samples, we compared the ability of these four models to fit the experimental data. Each model was globally fitted, with all kinetic parameters shared across initial monomer concentrations. The fits are shown in Figure S8, and the values of the fitted parameters and diagnostic statistics are shown in Table S3.

Firstly, we tested the Oosawa model (*19*). In this model, fibrils form by primary nucleation, which behaves as an n_c^{th}-order multimerization process. Fibrils then grow by stepwise addition of monomers to the fibril end, which behaves as a second-order association between a fibril and a free monomer. This model admits the exact solution (*13*, *20*)

$$\frac{M(t)}{m(0)} = 1 - \operatorname{sech}^{2/n_c}\left(\sqrt{\frac{n_c}{2}}\lambda t\right), \tag{S2}$$

where

$$\lambda = \sqrt{2k_n k_+ m(0)^{n_c}}. \tag{S3}$$

Here, k_n is the rate parameter for primary nucleation and k_+ is the rate parameter for elongation. As shown in Figure S8(a), the Oosawa model results in a poor fit. Specifically, the lack of a secondary process means the model cannot produce exponential early-time scaling, and lacks a pronounced lag phase. This agrees with our observation of exponential early-time kinetics, and the findings of previous work (*16*).

Next, we tested models that included a secondary process. Three such processes were considered: fragmentation (*13*, *14*, *21*), single-step secondary nucleation (*14*), and saturable multi-step secondary nucleation (*17*, *18*). In all three cases, the approximate solution has the same mathematical form, and the models differ only in the definitions of certain parameters. The general equation is (*13*, *14*, *21*)

$$\frac{M(t)}{m(0)} = 1 - \left(\frac{B_+ + C_+}{B_+ + C_+ e^{\kappa t}} \frac{B_- + C_+ e^{\kappa t}}{B_- + C_+}\right)^{\frac{k_\infty^2}{\kappa \tilde{k}_\infty}} e^{-k_\infty t}, \tag{S4}$$

where

$$B_{\pm} = \frac{k_{\infty} \pm \tilde{k}_{\infty}}{2\kappa}, \tag{S5}$$

$$C_{\pm} = \pm \frac{\lambda^2}{2\kappa^2}, \tag{S6}$$

$$\tilde{k}_{\infty} = \sqrt{k_{\infty}^2 - 4C_+C_-\kappa^2}. \tag{S7}$$

Here, λ has the same definition as Eq. (S3), and κ and k_{∞} have model-specific definitions. Since fragmentation can be treated as a special case of secondary nucleation (*14*, *16*), we begin with the definitions for single-step secondary nucleation,

$$k_{\infty} = \sqrt{\frac{2\kappa^2}{n_2(n_2+1)} + \frac{2\lambda^2}{n_c}}, \tag{S8}$$

$$\kappa = \sqrt{2k_2k_+m(0)^{n_2+1}}, \tag{S9}$$

where k_2 is the $(n_2+1)^{\text{th}}$-order rate constant for formation of a secondary fibril nucleus of effective size n_2, at a rate also proportional to the concentration of monomers already incorporated into fibrils (*14*).

The correct solution for fragmentation can be obtained by taking the limit $n_2 \to 0$ (in practice, setting $n_2 = 10^{-3}$) and substituting (*13*, *14*, *21*)

$$\kappa = \sqrt{2k_fk_+m(0)}, \tag{S10}$$

where k_f is the rate constant for fragmentation at each possible break point along the length of the fibril. When fitting the fragmentation-dominated model, the value of k_f was constrained such that $k_fk_+ = k_nk_+ \times (10^{-5.5}\ \text{M})^{n_c}/(10^{-8}\ \text{M})$, a constraint that was also applied in (*16*). This ensures that fragmentation becomes dominant when the fibril mass concentration $M(t) \approx 10$ nM for a reaction with initial monomer concentration $m(0) = \sqrt{10}$ µM, forcing the fitting algorithm to maintain an appreciable level of fragmentation. Without this constraint, k_f is simply minimized in order to attain a high concentration-dependence,

resulting in a fit very similar to that obtained for the Oosawa model (*16*).

For multi-step secondary nucleation, Meisl *et al.* (*17*) showed that Eq. (S4) can still be used with the modified definitions

$$\kappa = \sqrt{2k_2 k_+ m(0)^{n_2+1} \frac{K_2^{n_2}}{K_2^{n_2} + m(0)^{n_2}}}. \tag{S11}$$

$$k_\infty = \sqrt{A(\infty) - A(0)}, \tag{S12}$$

$$\begin{aligned} A(t) = &- \frac{\lambda^2}{n_c} - 2k_2 k_+ K_2 m(0) \frac{\log\left[K_2 + m(t)^{n_2}\right]}{n_2} \\ &- 2k_2 k_+ K_2 m(t) \left({}_2F_1\left[\frac{1}{n_2}, 1, 1 + \frac{1}{n_2}, -\frac{m(t)^{n_2}}{K_2}\right] - 1 \right), \end{aligned} \tag{S13}$$

where K_2 is the effective Michaelis constant for secondary nucleation, and ${}_2F_1$ is a hypergeometric function. Note that, unlike traditional Michaelis-Menten kinetics, the rate of saturable secondary nucleation has an n_2^{th}-order rather than a linear dependence on $m(t)$ in the low concentration limit (*15*, *17*, *18*).

As shown in Figure S8(b), fragmentation succeeds in producing exponential early-time scaling and a pronounced lag phase, but also results in a weak concentration-dependence and an overly sharp approach to the maximum fluorescence intensity. The latter two issues are caused by the insensitivity of fragmentation to depletion of the free monomer, and also lead to a high fitted reaction order of primary nucleation (n_c = 5.23), an attempt to compensate for the weak concentration scaling of the secondary process. Had the value of k_f been left unconstrained, the fit would instead have done this by minimising the role of the concentration-insensitive secondary pathway, producing a fit similar to Figure S8(a). Single-step secondary nucleation, the fit for which is shown in Figure S8(c), results in a much better fit, as it is able to simultaneously produce exponential early-time scaling, the correct concentration-dependence at intermediate $m(0)$, and a less abrupt approach to the maximum fluorescence intensity, due to its greater sensitivity to monomer depletion. Nonetheless, the fit is not perfect, since the concentration-dependence of the experimental data varies

slightly across the 1.5-6.0 µM range. Saturable secondary nucleation, shown in Figure S8(d), addresses this issue by allowing γ to vary with $m(0)$, and so further improves the fit. Although the difference between non-saturable and saturable secondary nucleation visually appears to be small, it is significant. Comparison of the two fits using Akaike's corrected information criterion (AICc) favored the latter (ΔAICc = 14736, equivalent to >99.99% probability).

Note that, in all the fits described above, the reaction orders n_c and n_2 were allowed to take non-integer values, reflecting the fact that they are effective reaction orders that reflect a variety of different possible nucleation pathways involving oligomers of diverse sizes. We also used the constraint $n_c = n_2$, which reduces the size of parameter space and is mechanistically justified, since secondary nucleation is currently believed to involve a similar mechanism to primary nucleation (*22*, *23*). Akaike's corrected information criterion (AICc) values were calculated from the residual sum of squares (RSS) according to the relation (*24*)

$$\mathrm{AICc} = N \ln\left(\frac{\mathrm{RSS}}{N}\right) + 2K + \frac{2K^2 + 2K}{N - K - 1}, \tag{S14}$$

where N is the number of data points and K is the number of fitted parameters. The likelihood of each model was proportional to $\exp\left(-\mathrm{AICc}/2\right)$, so that the relative likelihood between two models was $\exp\left(-\Delta\mathrm{AICc}/2\right)$. The fitted parameter values and diagnostic statistics are presented in Table S3.

Although saturable secondary nucleation provides the best fit for the data, the fit is still not perfect, with some discrepancies between the data and the fitted curves in the early growth phase, particularly at lower $m(0)$. To test whether this is caused by insufficiently exponential early-time scaling in the experimental data, or simply inaccurate scaling of λ and κ due to the occurrence of a more complex nucleation mechanism than the model is able to capture, we fitted Eq. (S4) individually to each Aβ(1-42) concentration, allowing λ and κ to deviate from the expected power-law or Michaelis-Menten-like scaling across $m(0)$ values. In effect, this tests whether Eq. (S4) has the correct functional form to describe the

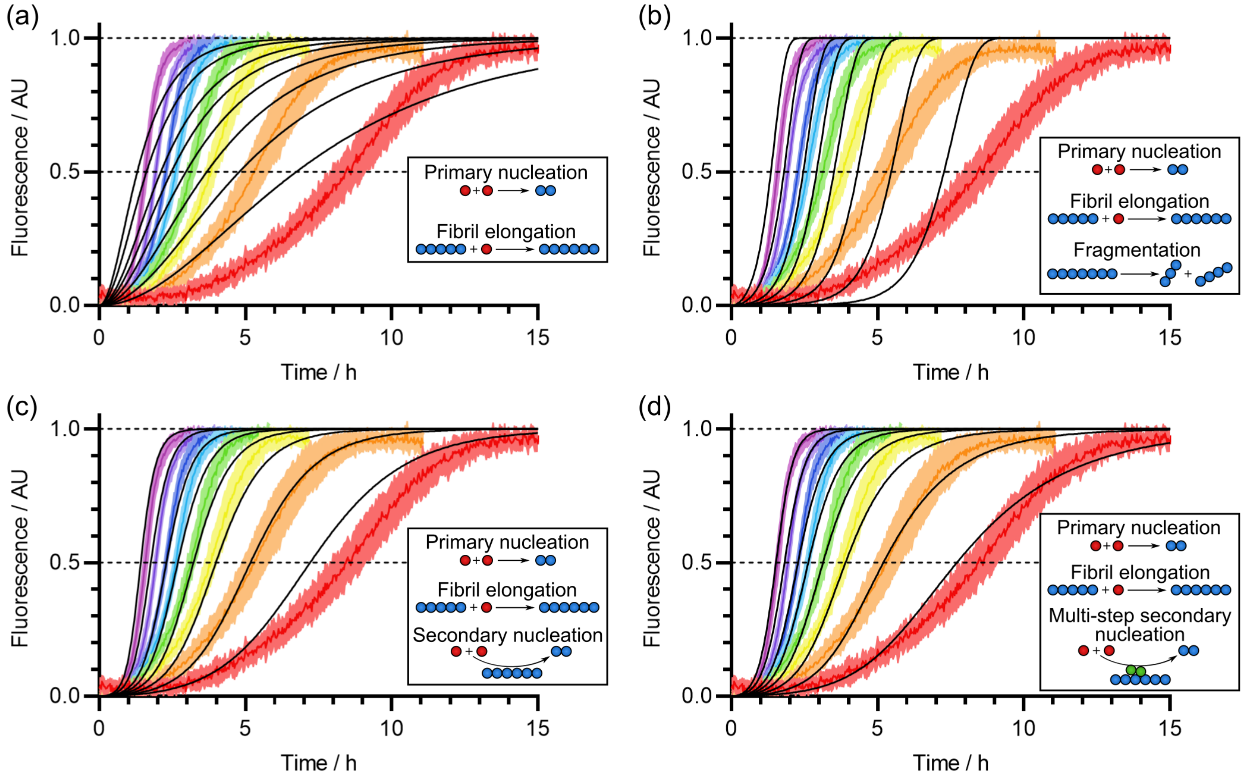

Figure S8: Comparison of global fitting of the self-assembly kinetics of commercial recombinant Aβ(1-42) solubilized by sonication for 5 min in 50 mM NaOH. The models are: (a) the Oosawa model, ie. basic nucleated polymerization in which primary nucleation and elongation are the only microscopic processes (*19*, *25*); (b) nucleated polymerization with fragmentation acting as the secondary process (*13*, *14*, *21*); (c) nucleated polymerization with single-step secondary nucleation (*14*); and (d) nucleated polymerization with saturable secondary nucleation (*17*, *18*). In each case, all fitted parameters are shared globally across Aβ(1-42) concentrations. The data fitted here are the same dataset that was presented in Figure 9 in the main text. The color scheme is the same across all panels, and indicates the initial Aβ(1-42) concentration: red, 1.5 µM; orange, 2.0 µM; yellow, 2.5 µM; green, 3.0 µM; cyan, 3.5 µM; blue, 4.0 µM; indigo, 5.0 µM; violet, 6.0 µM. The fitted curves are shown as black lines. The corresponding parameters are shown in Table S3.

Table S3: Best-fit parameters and diagnostic statistics for the global fits in this section. In cases where an n_2 value was present, the fit was constrained such that $n_c = n_2$, as in Refs. (*16*, *26*). As described in the text, the value of k_f was constrained to ensure that $k_f k_+ = k_n k_+ \times (10^{-5.5}\ \mathrm{M})^{n_c}/(10^{-8}\ \mathrm{M})$, as previously performed in Ref. (*16*).

**Parameter**	**Parameters from global fits**			
	**Primary only**	**Fragmentation**	**Secondary nucleation**	**Saturable secondary nucleation**
	**Fig. S9(a)**	**Fig. S9(b)**	**Fig. S9(c)**	**Fig. S9(d)**
n_c	2.37	5.23	1.68	2.37
n_2	—		1.68	2.37
$k_n k_+$ / $\mathrm{M}^{-n_c}.\mathrm{s}^{-2}$	9.72×10^4	2.43×10^{19}	1.75×10^1	1.19×10^4
$k_2 k_+$ / $\mathrm{M}^{-(n_2+1)}.\mathrm{s}^{-2}$	—		8.10×10^7	1.29×10^{12}
$k_f k_+$ / $\mathrm{M}^{-1}.\mathrm{s}^{-2}$	—	*see text*	—	
K_2 / µM	—			2.74
AICc	-125352	-126594	-166419	-181155
R^2	0.888	0.892	0.970	0.982

self-assembly of commercial recombinant Aβ(1-42) when λ and κ are allowed to vary more freely with $m(0)$. To reduce the size of parameter space, we set $n_c = n_2 = 2$ for this analysis. Individual fitting substantially improved fit quality, indicating that the remaining discrepancies in Figure S8(d), which are comparable to those seen in other papers using the Knowles model (*17*, *18*, *26–30*), do not mean that the wrong type of scaling is present in the early time. Instead, they indicate that the fitting algorithm is sacrificing fit quality in the early time for other regions of the curve. This may indicate that other processes are also present that still have not been accounted for, such as lag-phase oligomerization. However, the main conclusions are not affected: (i) the exponential early-time scaling supports the existence of a secondary process; (ii) the overall curve shape and concentration-dependence suggest a higher-order secondary process such as secondary nucleation, rather than a lower-order process such as fragmentation; and (iii) the concentration-dependence shows some evidence of variation with $m(0)$, which may be explained by saturation of secondary nucleation, or another process along the secondary pathway. Thus, the same conclusions can be drawn from this comparison of fits as were drawn in Cohen *et al.* (*16*).

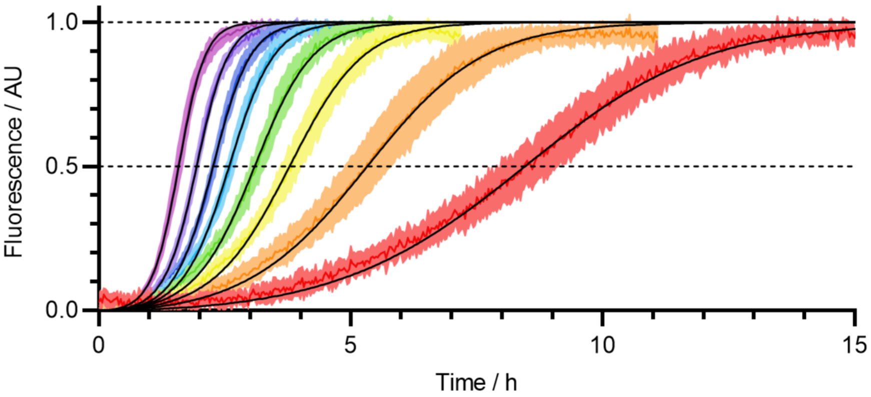

Figure S9: Individual fitting of Eq. (S4) to the self-assembly kinetics of commercial recombinant Aβ(1-42), showing that the equation has the correct functional form, and the remaining discrepancies in Figure S8(d) are due to inaccurate scaling of λ and κ. The representative data fitted here are the same dataset that was presented in Figure 9 in the main text. The color scheme is the same across all panels, and indicates the initial Aβ(1-42) concentration: red, 1.5 µM; orange, 2.0 µM; yellow, 2.5 µM; green, 3.0 µM; cyan, 3.5 µM; blue, 4.0 µM; indigo, 5.0 µM; violet, 6.0 µM. The fitted curves are shown as black lines.

S6 Re-freezing of Aβ solubilized in 50 mM NaOH

We carried out experiments to determine whether the remainder of Aβ(1-42) aliquots that had been thawed for use could be re-frozen, allowing Aβ(1-42) from a single aliquot to be used on multiple separate occasions. Aliquots that had been prepared by sonication for 5 min in 50 mM NaOH were thawed and then re-frozen according to one of three methods: rapid freezing by immersion in liquid N_2 (freezing time < 1 s); freezing by placing the Eppendorf tube in contact with pre-cooled metal at -80°C (< 1 min); and simply placing the sample in a rack in the -80°C freezer (< 5 min). While the former was obviously preferable, the other two methods were tested due to their convenience, and to ascertain whether improper freezing protocols could affect the quality of Aβ(1-42) aliquots. The AF4-MALS elugrams and ThT self-assembly kinetics obtained with re-frozen peptide, shown in Figure S10, are indistinguishable the results obtained with first-use aliquots (Figures 3-4 in the main text), and do not show a clear dependence on the re-freezing method. Therefore, Aβ(1-42) aliquots solubilised according to our recommended protocol can be re-frozen for multiple uses, which is convenient when performing a range of experiments requiring different quantities of peptide. Although the re-freezing protocol does not appear to make a difference, so long as the final temperature is -80°C, use of liquid N_2 is still recommended whenever possible.

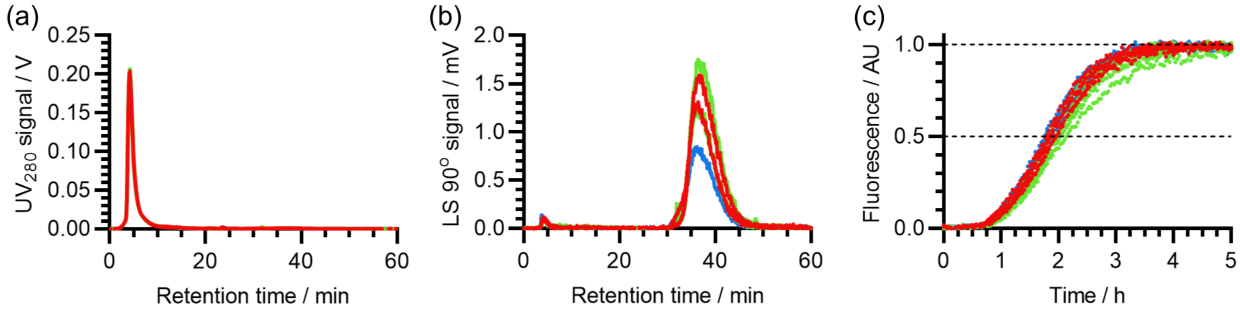

Figure S10: Effect of re-freezing on Aβ(1-42) solubilised by 5 min sonication in 50 mM NaOH. (a) UV_{280} signal from AF4-MALS separation of re-frozen Aβ(1-42) preparations. (b) The corresponding LS 90° signal. (c) The normalized ThT self-assembly kinetics. All panels use the same color scheme, which encodes the re-freezing method: red, placement in a rack in the -80°C freezer; green, freezing by placement in contact with pre-cooled metal at -80°C; blue, rapid freezing by immersion in liquid N_2.

S7 Absorbance spectroscopy of tyrosine and Aβ(1-42)

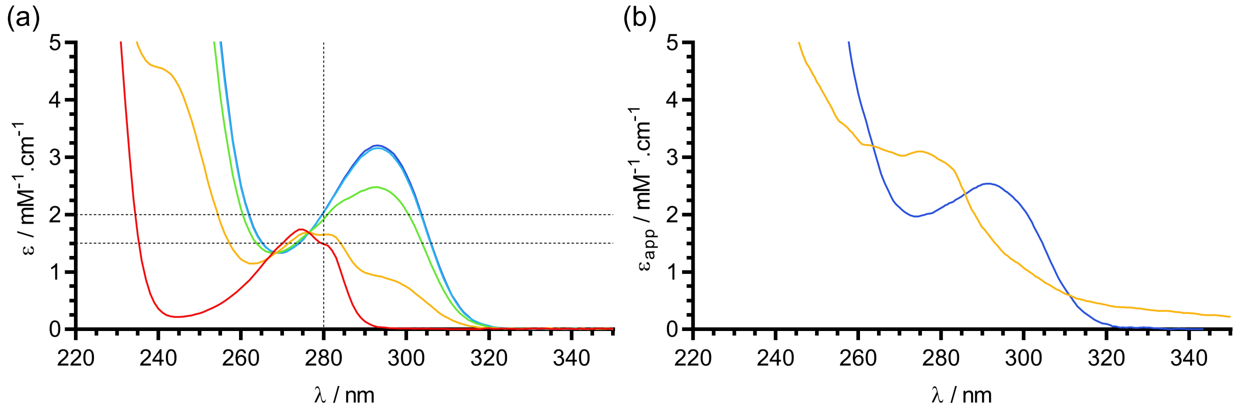

Figure S11: UV absorbance spectra of tyrosine and Aβ(1-42). (a) Tyrosine at varying pH: red, 7.0; amber, 10.0; green, 11.0; cyan, 12.0; blue, 12.7. While the protonated tyrosine sidechain (red) has a peak at 275, with a shoulder on the right-hand side, the deprotonated tyrosinate sidechain (blue) has a red-shifted peak with a maximum around 293 nm. As shown by the intercepts between the absorbance curves and the dashed vertical line, although there is an isobestic point at $\sim$276 nm, there is still an increase in extinction coefficient at 280 nm between pH 7.0 and 12.7. The horizontal dashed lines indicate intercepts with $\epsilon = 1500$ $M^{-1}.s^{-1}$ and $\epsilon = 2000$ $M^{-1}.s^{-1}$, and are included to guide the eye. (b) Aβ(1-42) resolubilized to a nominal concentration of 1 mg/ml by the two main protocols examined in this study: amber, sonication for 30 min in 10 mM NaOH (final pH 10.0); and blue, sonication for 5 min in 50 mM NaOH (final pH 12.5). Note that both spectra, but particularly the pH 10.0 spectrum, exhibit enhanced absorbance at short wavelengths compared to the spectra at similar pH in panel (a), a consequence of light scattering. The light scattering in the pH 10.0 sample reflects the extensive pre-aggregation revealed by AF4-MALS, whereas the less severe light scattering in the pH 12.5 sample is probably due to the HMW species that eluted after cross-flow in the AF4-MALS runs of those samples, as large quantities of oligomers were not found to be present. Note also that the absorbance peak of the pH 12.5 sample is 20% lower than that of the same nominal concentration of tyrosine in panel (a); this is probably due to experimental variation in the quantity of Aβ(1-42) in individual vials.

References

1. Jarrett, J. T., Berger, E. P., and Lansbury, P. T. (1993) The Carboxy Terminus of the β Amyloid Protein Is Critical for the Seeding of Amyloid Formation: Implications for the Pathogenesis of Alzheimer's Disease. *Biochemistry 32*, 4693–4697.

2. Lührs, T., Ritter, C., Adrian, M., Riek-Loher, D., Bohrmann, B., Döbeli, H., Schubert, D., and Riek, R. (2005) 3D structure of Alzheimer's amyloid-β(1–42) fibrils. *Proc. Natl. Acad. Sci. U. S. A. 102*, 17342–17347.

3. Yun, S., Urbanc, B., Cruz, L., Bitan, G., Teplow, D. B., and Stanley, H. E. (2007) Role of Electrostatic Interactions in Amyloid β-Protein (Aβ) Oligomer Formation: A Discrete Molecular Dynamics Study. *Biophys. J. 92*, 4064–4077.

4. Yang, M., and Teplow, D. B. (2008) Amyloid β-Protein Monomer Folding: Free-Energy Surfaces Reveal Alloform-Specific Differences. *J. Mol. Biol. 384*, 450–464.

5. Lendel, C., Bjerring, M., Dubnovitsky, A., Kelly, R. T., Filippov, A., Antzutkin, O. N., Nielsen, N. C., and Härd, T. (2014) A Hexameric Peptide Barrel as Building Block of Amyloid-β Protofibrils. *Angew. Chem. Int. Ed. 53*, 12756–12760.

6. Xiao, Y., Ma, B., McElheny, D., Parthasarathy, S., Long, F., Hoshi, M., Nussinov, R., and Ishii, Y. (2015) Aβ(1–42) fibril structure illuminates self-recognition and replication of amyloid in Alzheimer's disease. *Nat. Struct. Mol. Biol. 22*, 499–505.

7. Wälti, M. A., Ravotti, F., Arai, H., Glabe, C. G., Wall, J. S., Böckmann, A., Güntert, P., Meier, B. H., and Riek, R. (2016) Atomic-resolution structure of a disease-relevant Aβ(1–42) amyloid fibril. *Proc. Natl. Acad. Sci. U. S. A. 113*, E4976–E4984.

8. Meral, D., and Urbanc, B. (2013) Discrete Molecular Dynamics Study of Oligomer Formation by N-Terminally Truncated Amyloid β-Protein. *J. Mol. Biol. 425*, 2260–2275.

9. Bayer, T. A., and Wirths, O. (2014) Focusing the amyloid cascade hypothesis on N-truncated Abeta peptides as drug targets against Alzheimer's disease. *Acta Neuropathol. 127*, 787–801.

10. Mital, M., Wezynfeld, N. E., Frączyk, T., Wiloch, M. Z., Wawrzyniak, U. E., Bonna, A., Tumpach, C., Barnham, K. J., Haigh, C. L., Bal, W., and Drew, S. C. (2015) A Functional Role for Aβ in Metal Homeostasis? N-Truncation and High-Affinity Copper Binding. *Angew. Chem. Int. Ed. 54*, 10460–10464.

11. Wulff, M., Baumann, M., Thümmler, A., Yadav, J. K., Heinrich, L., Knüpfer, U., Schlenzig, D., Schierhorn, A., Rahfeld, J.-U., Horn, U., Balbach, J., Demuth, H.-U., and Fändrich, M. (2016) Enhanced Fibril Fragmentation of N-Terminally Truncated and Pyroglutamyl-Modified Aβ Peptides. *Angew. Chem. Int. Ed. 55*, 5081–5084.

12. Walsh, D. M., Thulin, E., Minogue, A. M., Gustavsson, N., Pang, E., Teplow, D. B., and Linse, S. (2009) A facile method for expression and purification of the Alzheimer's disease-associated amyloid β-peptide. *FEBS J. 276*, 1266–1281.

13. Cohen, S. I. A., Vendruscolo, M., Welland, M. E., Dobson, C. M., Terentjev, E. M., and Knowles, T. P. J. (2011) Nucleated polymerization with secondary pathways. I. Time evolution of the principal moments. *J. Chem. Phys. 135*, 065105.

14. Cohen, S. I. A., Vendruscolo, M., Dobson, C. M., and Knowles, T. P. J. (2011) Nucleated polymerization with secondary pathways. II. Determination of self-consistent solutions to growth processes described by non-linear master equations. *J. Chem. Phys. 135*, 065106.

15. Dear, A. J., Meisl, G., Michaels, T. C. T., Zimmermann, M. R., Linse, S., and Knowles, T. P. J. (2020) The catalytic nature of protein aggregation. *J. Chem. Phys. 152*, 045101.

16. Cohen, S. I. A., Linse, S., Luheshi, L. M., Hellstrand, E., White, D. A., Rajah, L., Otzen, D. E., Vendruscolo, M., Dobson, C. M., and Knowles, T. P. J. (2013) Proliferation

of amyloid-β42 aggregates occurs through a secondary nucleation mechanism. *Proc. Natl. Acad. Sci. U. S. A. 110*, 9758–9763.

17. Meisl, G., Yang, X., Hellstrand, E., Frohm, B., Kirkegaard, J. B., Cohen, S. I. A., Dobson, C. M., Linse, S., and Knowles, T. P. J. (2014) Differences in nucleation behavior underlie the contrasting aggregation kinetics of the Aβ40 and Aβ42 peptides. *Proc. Natl. Acad. Sci. U. S. A. 111*, 9384–9389.

18. Meisl, G., Yang, X., Dobson, C. M., Linse, S., and Knowles, T. P. J. (2017) Modulation of electrostatic interactions to reveal a reaction network unifying the aggregation behaviour of the Aβ42 peptide and its variants. *Chem. Sci. 8*, 4352–4362.

19. Oosawa, F., and Kasai, M. (1962) A theory of linear and helical aggregations of macromolecules. *J. Mol. Biol. 4*, 10–21.

20. Flyvberg, H., Jobs, E., and Leibler, S. (1996) Kinetics of self-assembling microtubules: an "inverse problem" in biochemistry. *Proc. Natl. Acad. Sci. U. S. A. 93*, 5975–5979.

21. Knowles, T. P. J., Waudby, C. A., Devlin, G. L., Cohen, S. I. A., Aguzzi, A., Vendruscolo, M., Terentjev, E. M., Welland, M. E., and Dobson, C. M. (2009) An analytical solution to the kinetics of breakable filament assembly. *Science 326*, 1533–1537.

22. Ruschak, A. M., and Miranker, A. D. (2007) Fiber-dependent amyloid formation as catalysis of an existing reaction pathway. *Proc. Natl. Acad. Sci. U. S. A. 104*, 12341–12346.

23. Šarić, A., Buell, A. K., Meisl, G., Michaels, T. C. T., Dobson, C. M., Linse, S., Knowles, T. P. J., and Frenkel, D. (2016) Physical determinants of the self-replication of protein fibrils. *Nat. Phys. 12*, 874–880.

24. Burnham, K. P., and Anderson, D. R. *Model Selection and Multimodel Inference: A practical information-theoretic approach.*; Springer: Berlin, 2002.

25. Oosawa, F., and Asakura, S. *Thermodynamics of the Polymerization of Protein.*; Academic Press: New York, 1975.

26. Silvers, R., Colvin, M. T., Frederick, K. K., Jacavone, A. C., Lindquist, S., Linse, S., and Griffin, R. G. (2017) Aggregation and Fibril Structure of AβM01-42 and Aβ1-42. *Biochemistry 56*, 4850–4859.

27. Arosio, P., Cukalevski, R., Frohm, B., Knowles, T. P. J., and Linse, S. (2014) Quantification of the Concentration of Aβ42 Propagons during the Lag Phase by an Amyloid Chain Reaction Assay. *J. Am. Chem. Soc. 136*, 219–225.

28. Szczepankiewicz, O., Linse, B., Meisl, G., Thulin, E., Frohm, B., Frigerio, C. S., Colvin, M. T., Jacavone, A. C., Griffin, R. G., Knowles, T., Walsh, D. M., and Linse, S. (2015) N-Terminal Extensions Retard Aβ42 Fibril Formation but Allow Cross Seeding and Coaggregation with Aβ42. *J. Am. Chem. Soc. 137*, 14673–14685.

29. Yang, X., Meisl, G., Frohm, B., Thulin, E., Knowles, T. P. J., and Linse, S. (2018) On the role of sidechain size and charge in the aggregation of Aβ42 with familial mutations. *Proc. Natl. Acad. Sci. U. S. A. 115*, E5849–E5858.

30. Scheidt, T., Łapińska, U., Kumita, J. R., Whiten, D. R., Klenerman, D., Wilson, M. R., Cohen, S. I. A., Linse, S., Vendruscolo, M., Dobson, C. M., Knowles, T. P. J., and Arosio, P. (2019) Secondary nucleation and elongation occur at different sites on Alzheimer's amyloid-β aggregates. *Sci. Adv. 5*, eaau3112.
